# Induction of Ferroptosis in Glioblastoma and Ovarian Cancers by a New Pyrrole Tubulin Assembly Inhibitor

**DOI:** 10.1021/acs.jmedchem.2c01457

**Published:** 2022-11-17

**Authors:** Michela Puxeddu, Jianchao Wu, Ruoli Bai, Michele D’Ambrosio, Marianna Nalli, Antonio Coluccia, Simone Manetto, Alessia Ciogli, Domiziana Masci, Andrea Urbani, Cinzia Fionda, Sonia Coni, Rosa Bordone, Gianluca Canettieri, Chiara Bigogno, Giulio Dondio, Ernest Hamel, Te Liu, Romano Silvestri, Giuseppe La Regina

**Affiliations:** † Laboratory Affiliated with the Institute Pasteur Italy - Cenci Bolognetti Foundation, Department of Drug Chemistry and Technologies, Sapienza University of Rome, Piazzale Aldo Moro 5, 00185Rome, Italy; ‡ Molecular Pharmacology Branch, Developmental Therapeutics Program, Division of Cancer Treatment and Diagnosis, Frederick National Laboratory for Cancer Research, National Cancer Institute, National Institutes of Health, Frederick, Maryland21702, United States; § Department of Basic Biotechnological Sciences, Intensivological and Perioperative Clinics, Catholic University of the Sacred Heart, Largo Francesco Vito 1, 00168Rome, Italy; ∥ Laboratory Affiliated with the Institute Pasteur Italy - Cenci Bolognetti Foundation, Department of Molecular Medicine, Sapienza University of Rome, Viale Regina Elena 291, 00161Rome, Italy; ⊥ Aphad SrL, Via della Resistenza 65, 20090Buccinasco, Italy; # Shanghai Geriatric Institute of Chinese Medicine, Shanghai University of Traditional Chinese Medicine, 365 South Xiangyang Road, 200031Shanghai, China

## Abstract

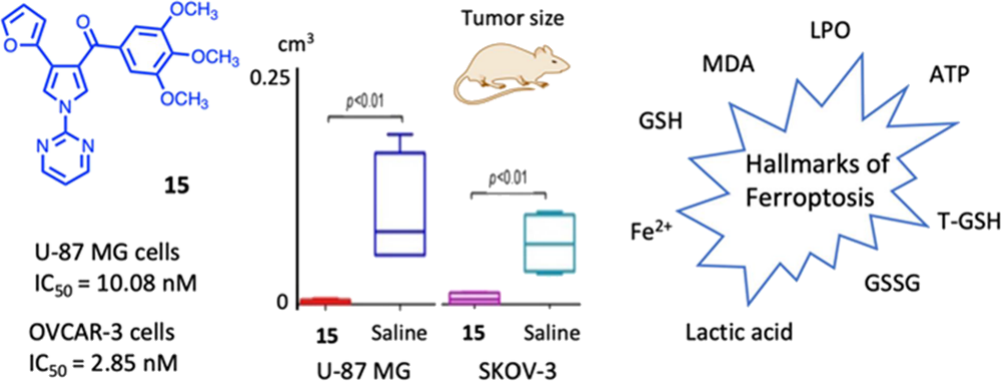

We synthesized new aroyl diheterocyclic pyrrole (ARDHEP) **15** that exhibited the hallmarks of ferroptosis. Compound **15** strongly inhibited U-87 MG, OVCAR-3, and MCF-7 cancer cells, induced an increase of cleaved PARP, but was not toxic for normal human primary T lymphocytes at 0.1 μM. Analysis of the levels of lactoperoxidase, malondialdehyde, lactic acid, total glutathione, and ATP suggested that the in vivo inhibition of cancer cell proliferation by **15** went through stimulation of oxidative stress injury and Fe^2+^ accumulation. Quantitative polymerase chain reaction analysis of the mRNA expression in U-87 MG and SKOV-3 tumor tissues from **15**-treated mice showed the presence of *Ptgs2*/*Nfe2l2*/*Sat1*/*Akr1c1*/*Gpx4* genes correlated with ferroptosis in both groups. Immunofluorescence staining revealed significantly lower expressions of proteins Ki67, CD31, and ferroptosis negative regulation proteins glutathione peroxidase 4 (GPX4) and FTH1. Compound **15** was found to be metabolically stable when incubated with human liver microsomes.

## Introduction

Glioblastoma multiforme (GBM), a grade IV glioma and one of the most aggressive forms of malignancy, arises within the brain and infiltrates rapidly into adjacent brain tissue.^1,2^ GBM represents 45% of malignant tumors of the brain and the central nervous system.^3^ The poor prognosis persists following standard treatments with surgery, radiotherapy, and chemotherapy. Patients with GBM tumors have only a 14 month life expectancy from diagnosis and are difficult to treat. They tend to relapse and show drug resistance to current therapy.^2,3^ Standard chemotherapy of GBM includes temozolomide, a mustard that does significantly increase survival when combined with radiotherapy. Bevacizumab, an anti-VEGF monoclonal antibody (mAb), was approved for the treatment of recurrent GBM. Carmustine is a nitrosourea in use for the treatment of both recurrent and newly diagnosed GBM, but it causes severe bone marrow, liver, and kidney toxicity.^4,5^

Ovarian cancer (OC) is the most frequent cause of death among gynecologic cancers and the deadliest malignancy in women. NIH cancer statistics for 2021 estimated 21,410 new cases (1.1% of all new cancer cases) and 13,770 deaths. The 5 year relative survival for 2011–2017 is 49%.^6^ Debulking surgery and radiation therapy are the standard treatment for nonmetastatic disease. Depending on the type of OC, different therapeutic managements are used. Chemotherapy with tubulin binding agents, mustards, and intercalating agents are standard components of OC treatment. Advanced level treatment options may also include targeted therapy, immunotherapy, and hormone therapy.^7,8^

After the first-line treatment, GMB tumors progress with limited treatment options. Current systemic therapy with temozolomide and nitrosoureas has limited efficacy, and re-surgery or re-irradiation may be useful in selected cases. Positive therapeutic responses to recurrent GMB can be observed in a highly selected and very limited patient population.^9^ Standard chemotherapy of recurrent OC with tubulin binding/platinum agents significantly improved progression-free survival up to 15 months after the introduction of the mAb bevacizumab.^10^ However, nearly 23% of patients relapse within 6 months after the end of primary chemotherapy and 60% relapse after a further 6 months.^11,12^ Bevacizumab also has been used extensively to treat recurrent GMB in patients who have failed the first line therapy.^13^ However, despite a high initial response rate, the effect is transient and the tumors of most patients progress rapidly.^14,15^

Apoptosis (programmed cell death) has been long recognized as critical for sustained tumor suppression following anticancer treatments. Recently, ferroptosis is being increasingly investigated as nonapoptotic, iron-dependent regulated cell death (RCD), distinct from other forms, such as necroptosis, pyroptosis, and alkaliptosis, with potential to overcome the block of apoptosis in some cancer cells.^16,17^ The ferroptotic pathway is mainly triggered by peroxidation of extra-mitochondrial lipid arising from the accumulation of iron-dependent reactive oxygen species (ROS) The induction of ROS production in most tumors follows a state of high oxidative stress caused by excessive iron derived from abnormalities of two major redox systems, lipid peroxidation and thiols, and from aberrant iron metabolism.^18^ Many cancers are ferroptosis-related.^19^ Therefore, inducing ferroptosis can be an effective approach to eradicate residual or resistant cancer cells.^20^ Much evidence also supports the idea of inducing ferroptosis in GBM therapies.^21^ Moreover, OC cells have shown susceptibility to ferroptosis because excess iron can overload tumor-initiating cells following overexpression of transferrin receptor 1 and a decrease in the level of the iron efflux pump ferroportin.^22^

**Chart 1:**
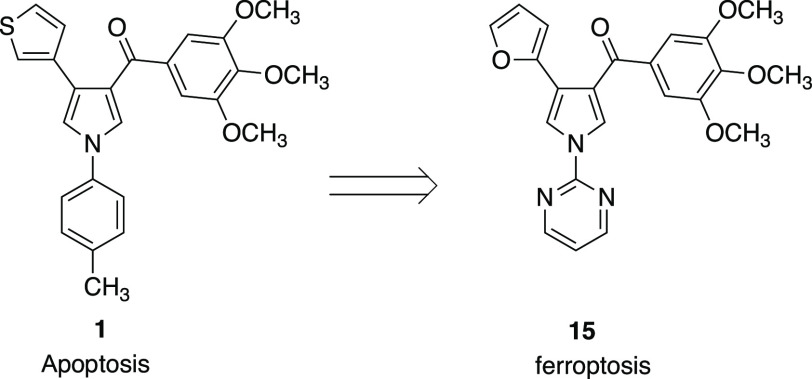
Structures of Compounds **1** and **15**

In this work, we replaced the 1-(methylphenyl) group of **1**(23) with a pyridine or pyrimidine ring and kept fixed the hydrogen, phenyl, or furan-2-yl moiety at position 4 of the pyrrole. The latter heterocyclic ring has shown a tight interaction with the colchicine site of tubulin.^24^ We found that a new aroyl diheterocyclic pyrrole (ARDHEP) derivative, **15**, whose mechanism is the inhibition of tubulin polymerization, exhibited the hallmarks of ferroptosis rather than the conventional apoptosis found with **1** (Chart 1). However, angiogenic effects were similar for both compounds. In this paper, we report the synthesis of **15** and its antitumor activity in vitro and in vivo, as well as its selectivity for cancer cells as compared with normal human cells.

## Results and Discussion

### Chemistry

#### Synthesis of Compounds **2–16**

**Scheme 1:**
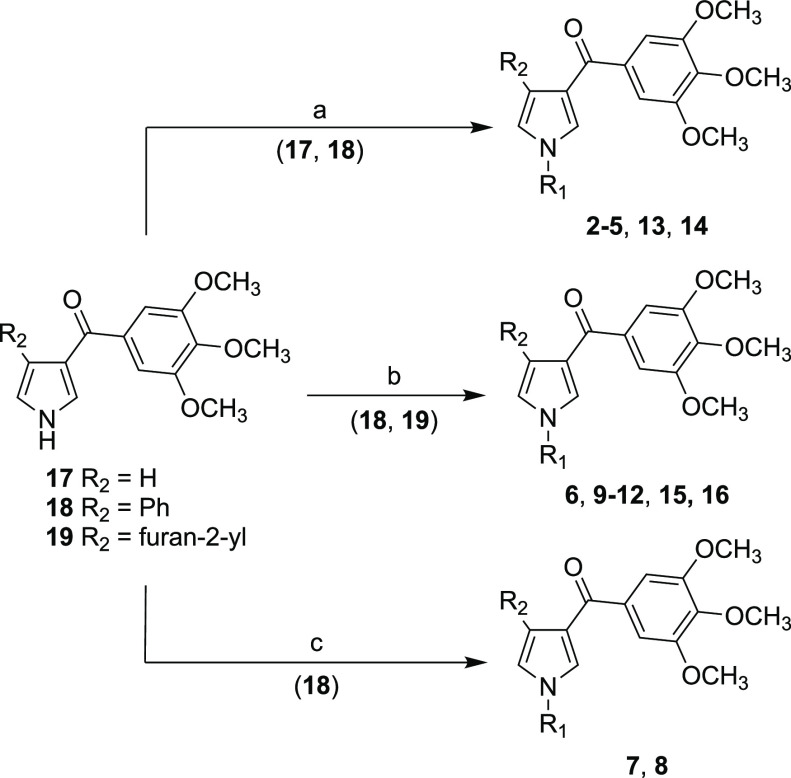
R_1_ = Pyridin-2-yl, Pyridin-3-yl, Pyridin-4-yl, Pyrimidin-2-yl, Pyrimidin-5-yl, Pyrazin-2-yl, R_2_ = H, Phenyl, Furan-2-yl^a^ Reagents and reaction conditions: (a) (**2–5**, **13**) appropriate halo-heterocycle, copper(I) iodide, cesium carbonate, 1,10-phenanthroline, 1,4-dioxane, closed vessel, 250 W, 220 °C, 25 min, 23–84%; (b) (**6**, **9–12**, **15**, **16**) appropriate halo-heterocycle, copper(I) iodide, cesium carbonate, 1,10-phenanthroline, 1,4-dioxane, 100 °C, 24 h, argon stream, 8–99%; (c) (**7**, **8**) appropriate boronic acid, copper(II) acetate, triethylamine, 1,2-dichloroethane, 40 °C, 18 h, argon stream, 12 and 9%.

Treatment of the aroyl pyrroles **17–19**(23,24) with the appropriate halo-heterocycle in the presence of copper(I) iodide, cesium carbonate, and 1,10-phenanthroline in 1,4-dioxane under microwave (MW) irradiation in a closed vessel at 220 °C for 25 min or at 100 °C for 24 h under an argon stream furnished, respectively, compounds **2–5** and **13**, **14** (Scheme 1, method a) and **6**, **9–12**, **15**, and **16** (Scheme 1, method b). Compounds **7** and **8** were prepared by reacting aroyl pyrrole **18** with the appropriate pyrinylboronic acid in the presence of copper(II) acetate and triethylamine in 1,2-dichloroethane at 40 °C for 18 h under an argon stream (Scheme 1, method c). Compound **17** was synthesized by sodium hydroxide hydrolysis of (1-tosyl-1*H*-pyrrol-3-yl)(3,4,5-trimethoxyphenyl)methanone in aqueous ethanol under MW irradiation.^24^ Compounds **18** and **19** were prepared from 1-phenyl-3-(3,4,5-trimethoxyphenyl)prop-2-en-1-one or 3-(furan-2-yl)-1-(3,4,5-trimethoxyphenyl)prop-2-en-1-one and *p*-toluenesulfonylmethyl isocyanide in dimethyl sulfoxide/diethyl ether, respectively, in the presence of sodium hydride, as previously described.^23^

### Biology

#### Inhibition of Cancer Cell Growth

##### MCF-7 Cells

Inhibition of the growth of human breast carcinoma (MCF-7) cancer cells was strongly correlated with the nature of the heterocycle at position 1 of the pyrrole and the R_2_ substituent at the 4 position (Table 1). Compounds **2–5** without the R_2_ aromatic substituent were weak inhibitors of MCF-7 cancer cell growth, independent of the heterocycle at position 1 of the pyrrole nucleus. Among pyridines **6–11**, pyridin-2-yl **6** and **9** and pyridin-3-yl **7** and **10** derivatives inhibited cell growth with IC_50_’s of 18–50 nM, while pyridin-4-yl compounds **8** and **11** were weak inhibitors. Among compounds **13–16**, pyrimidin-2-yl **13** and **15** and pyrazin-2-yl **12**, derivatives with nitrogen atom(s) close to the carbon atom linked to the pyrrole nitrogen, showed strong inhibition of MCF-7 cell growth, while pyrimidin-5-yl derivatives **14** and **16** were poor inhibitors of cell growth. Introduction of the furan-2-yl at position 4 of the pyrrole tended to reinforce the inhibition of MCF-7 cell growth; in particular, derivative **15** exhibited the strongest inhibition within the series with an IC_50_ of 4.0 nM.

**Table 1:**
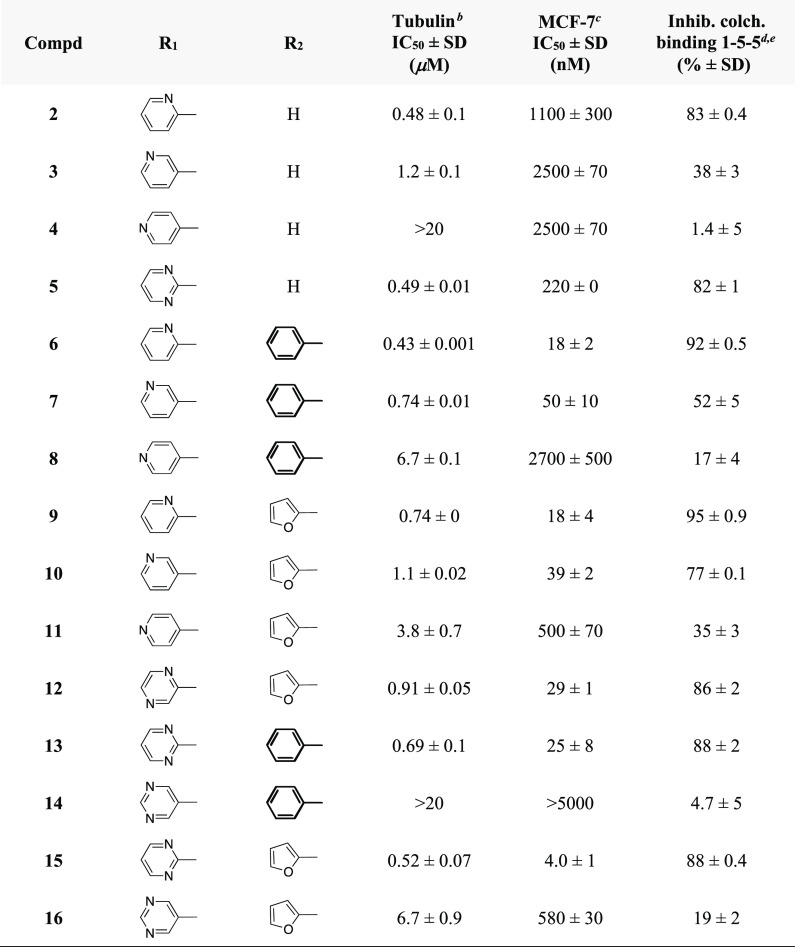
Inhibition of Tubulin Polymerization, Inhibition of the Growth of MCF-7 Human Breast Carcinoma Cells by Compounds **2–16**, and Inhibition of the Binding of Colchicine to Tubulin^*a*^

a Experiments were performed in duplicate or triplicate.

b Inhibition of tubulin polymerization. Tubulin was at 10 μM in the assembly assay. CSA4 as a reference compound yielded IC_50_s in the 0.54–0.73 mM range.

c Inhibition of the growth of MCF-7 human breast carcinoma cells.

d Inhibition of [^3^H]colchicine binding: tubulin, [^3^H]colchicine, inhibitor at 1:5:5 μM.

e Inhibition of [^3^H]colchicine binding: tubulin, [^3^H]colchicine, inhibitor at 1:5:1 μM: **6**, 69 ± 1%; **9**, 66 ± 2%. Inhibition of colchicine binding by CSA4: 98% at 5 μM, 78% at 1 μM.

##### U-87 MG Cells and OVCAR-3 Cells

**Figure 1:**
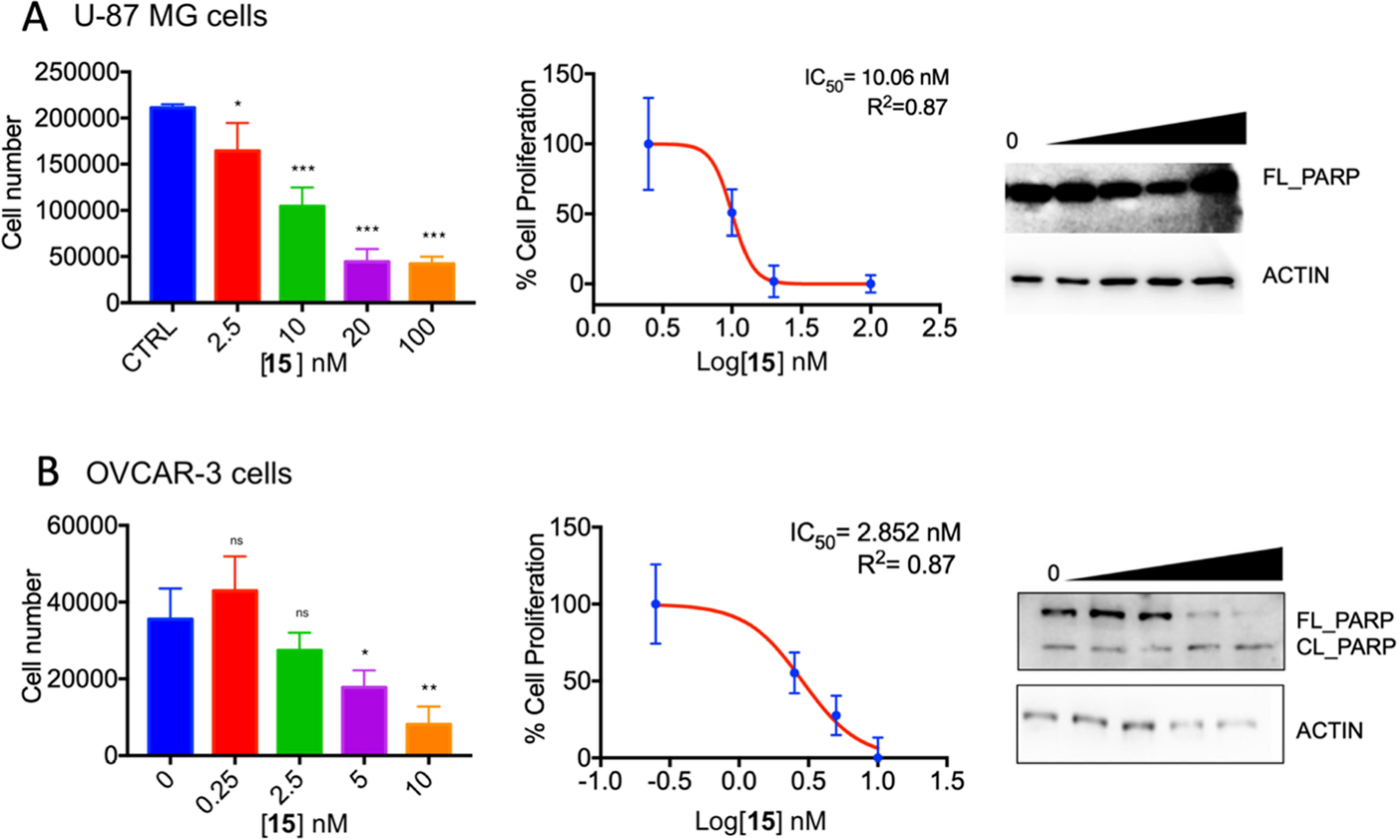
(A) Left panel, proliferation assay of U-87 MG GBM cells treated with increasing concentrations of compound **15** for 48 h. Experiment was performed in triplicate. Central panel, IC_50_ concentration of derivative **15** after a 48 h treatment of U-87 MG cells, calculated with Graphpad Prism 7.0 software. Experiment was performed in triplicate. Right panel, western blot of U-87 MG cells treated with increasing concentrations of compound **15** (0, 2.5, 10, 20, and 100 nM). PARP full length and cleaved is shown. Actin, loading control. (B) Left panel, proliferation assay of OVCAR-3 OC cells treated with increasing concentrations of compound **15** for 48 h. Experiment was performed in triplicate. Central panel, IC_50_ concentration of derivative **15** after a 48 h treatment of OVCAR-3 cells, calculated with Graphpad Prism 7.0 software. Experiment was performed in triplicate. Left panel, western blot of OVCAR-3 cells treated with increasing concentrations of compound **15** (0, 0.25, 2.5, 5, 10 nM). PARP full length and cleaved is shown. Actin, loading control. For both panels: statistical analysis was performed on three experiments: ns not significant, *p < 0.05, **p < 0.01, by one-way ANOVA test. Data represent the mean ± SD of one experiment performed in triplicate and repeated at least three times.

Upon incubation for 48 h, compound **15** inhibited in a dose-dependent manner the cell viability of human GBM U-87 MG cells with an IC_50_ of 10.06 nM and ovarian adenocarcinoma OVCAR-3 cells with an IC_50_ of 2.85 nM. Compound **15** was thus 3.5-fold less effective as an inhibitor of the U-87 MG cells as compared to the OVCAR-3 cell line (Figure 1A,B). To determine if the drug caused programmed cell death, we analyzed the cleavage of PARP by immunoblotting. As shown in Figure 1B, right panel, compound **15** induced a significant increase of cleaved PARP, starting from 5 nM. The proliferation of GBM cells U-87 MG was also inhibited by the same compound in a dose-dependent fashion, and this, too, was accompanied by the PARP cleavage, starting from 20 nM, consistent with their lower sensitivity to compound **15** as compared with the OVCAR-3 cells.

##### Human Primary T Lymphocytes

Potential toxicity on healthy cells was evaluated by treating human primary T lymphocytes with 0.1, 1.0, and 2.5 μM **15** or with control vehicle (dimethyl sulfoxide; DMSO). The frequency of early and late apoptotic cells was assayed by staining with annexin V and propidium iodide for 48 or 72 h. Compared to untreated or DMSO control, flow cytometric analysis showed that exposure to 0.1 μM **15** did not change cell viability, while some cytotoxicity was observed with **15** at 1.0 or 2.5 μM. These results indicated that this compound at 0.1 μM is not toxic for normal cells (Figures S1 and S2, Supporting Information).

#### Tubulin Polymerization Inhibition and [^3^H]Colchicine Binding

Compounds **5**, **13**, and **15** bearing the 1-(pyrmidin-2-yl) nucleus were strong inhibitors of tubulin polymerization with IC_50_ values of 0.49, 0.69, and 0.52 μM, respectively, compared with combretastatin A-4 (CSA4, IC_50_ = 0.54–0.73 μM range). Compounds **5**, **13**, and **15** inhibited the binding of [^3^H]colchicine to tubulin in the range of 82–88% with tubulin at 1 μM and the [^3^H]colchicine and inhibitor at 5 μM. Among tested compounds, **6** and **9** exhibited the best inhibition: 92 and 95%, and 66 and 69%, respectively, with tubulin and inhibitor at 1 μM and the [^3^H]colchicine at 5 μM (Table 1, footnote e).

#### Inhibition of In Vivo Growth of Cancer Cells by Stimulating Oxidative Stress Injury and Fe^2+^ Content

**Figure 2:**
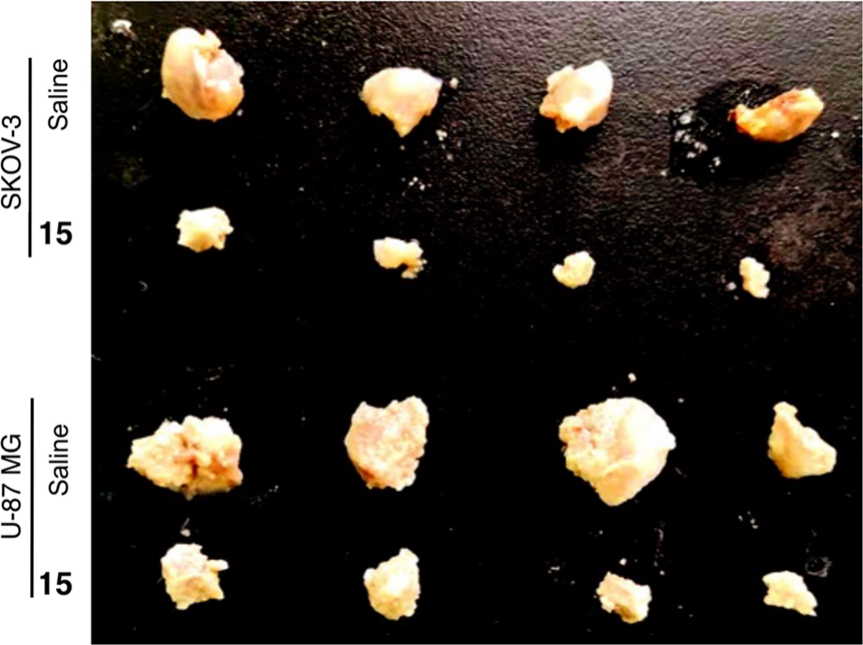
Tumor tissues: compound **15** inhibited the in vivo tumorigenicity of the human GBM cell line U-87 MG and the human OC cell line SKOV-3.

**Figure 3:**
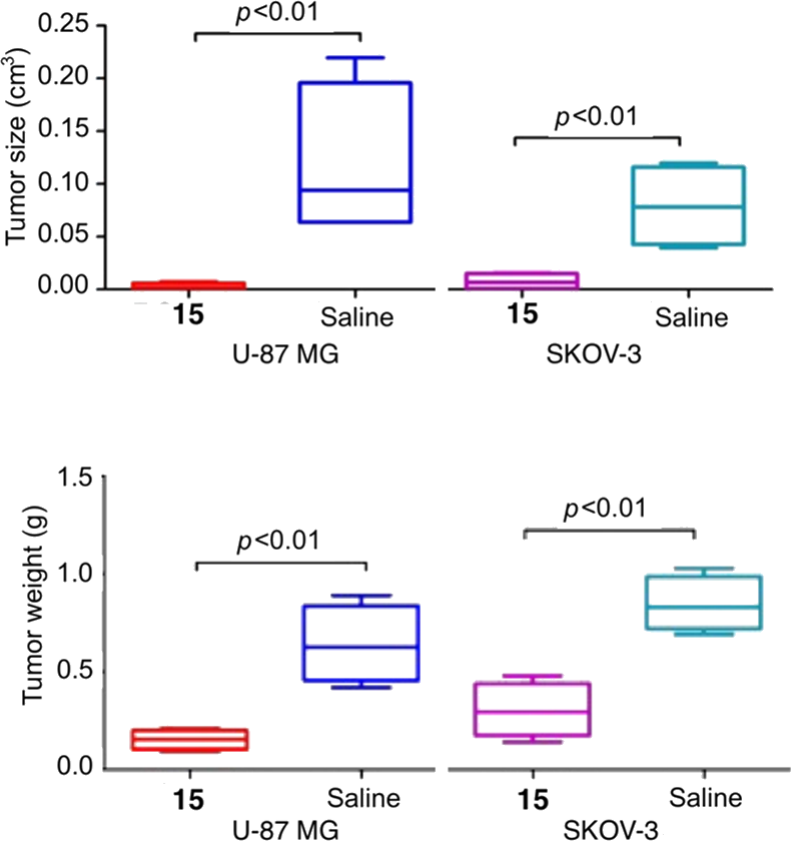
Weight and volume of tumors following treatment with compound **15** as compared to saline.

**Figure 4:**
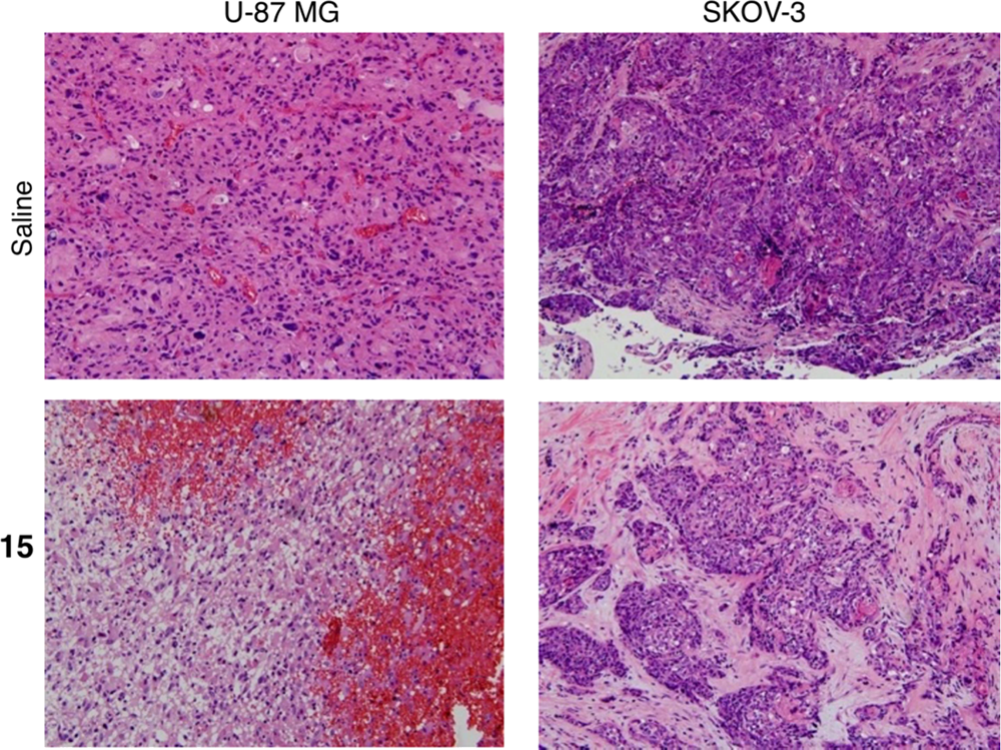
HE staining showed tumors were identical to the implanted tumors GBM or human OC. Magnification 200×.

BALB/C^nu/nu^ mice were inoculated subcutaneously with 1 × 10^8^ U-87 MG or SKOV-3 cells/mL and treated with intraperitoneal injection of 100 μL of **15** (25 mg/kg) every 2 days. At the same time, control groups of BALB/C^nu/nu^ mice were treated with intraperitoneal injection of 100 μL of saline every 2 days. Mice were euthanized on day 40, and tumors on their backs were collected for the measurement of tumor volume and weight. The tumors from the two groups treated with compound **15** were significantly smaller than the tumors in both control groups (Figures 2 and 3). Hematoxylin and eosin (HE) staining showed that the tumor types were identical to those originally implanted (Figure 4).

**Figure 5:**
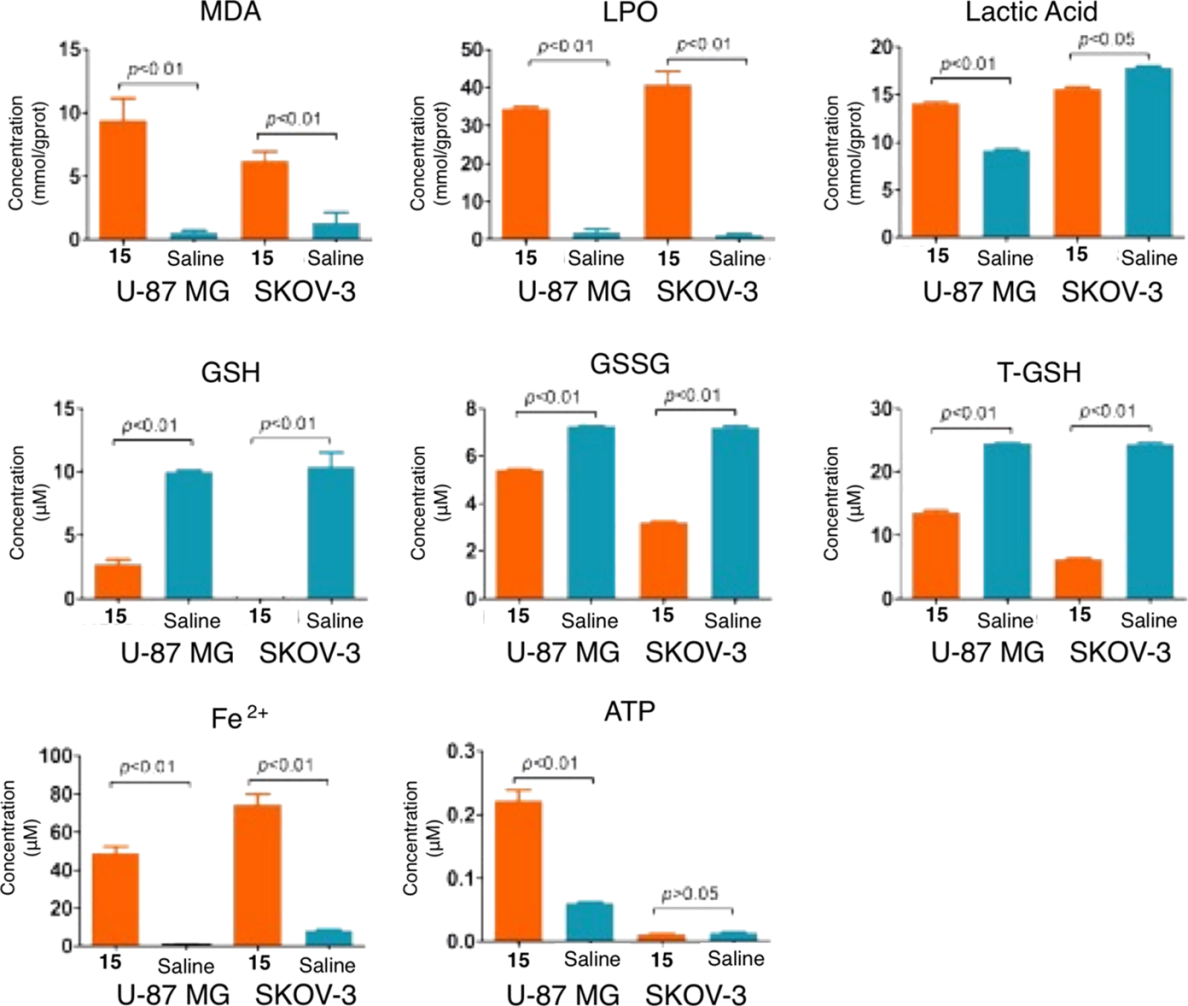
Quantitation of oxidative stress-related enzymes, metabolites, ATP, and Fe^2+^ derived from harvested tumors.

Biochemical analysis showed that levels of lactoperoxidase (LPO), a heme-containing mammalian peroxidase, malondialdehyde (MDA), an end product of lipid peroxidation, and lactic acid in tumor tissues derived from the **15**-treated groups were appreciably higher than those in the control groups (Figure 5, bottom panel). However, the levels of total glutathione (T-GSH) [glutathione (GSH) + oxidized glutathione (GSSG)] and ATP were significantly lower in tumor tissues derived from the **15**-treated groups than those in the control groups (Figure 5, bottom panel).

Cell death in GSH-depleted cells has been described as occurring through ferroptosis and autophagy and that ferroptosis is a primary mechanism of GSH depletion-induced cell death in retinal pigment epithelial cells.^25^ We also found that the concentrations of Fe^2+^ in tumor tissues derived from the **15** treatment groups were significantly higher than those in the control groups (Figure 5). The presence of sufficient free intracellular iron and the presence of membrane oxidizable phospholipids acylated with polyunsaturated fatty acids are both prerequisites for the occurrence of ferroptosis.^26^ Thus, our results are consistent with the conclusion that because **15** caused oxidative stress injury and Fe^2+^ accumulation, in vivo inhibition of proliferation of the cancer cells was caused by ferroptosis.

#### Gene Expression Profiles of Proliferation and Ferroptosis in Tumor Tissues by Compound **15**

**Figure 6:**
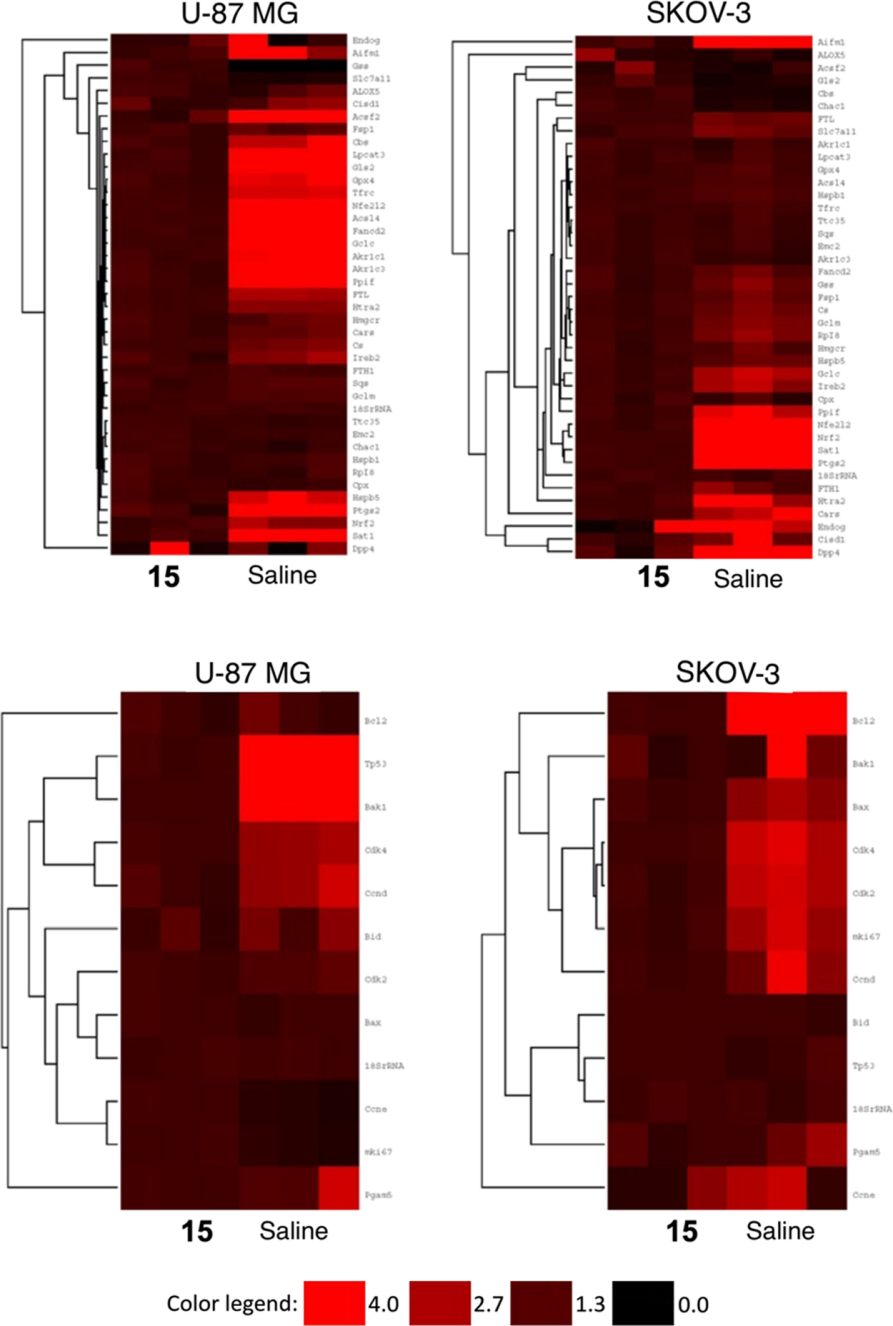
qPCR results of differential gene expression profiles of ferroptosis in each tumor tissue (top panel). qPCR results of differential gene expression profiles of proliferation in each tumor tissues (bottom panel).

Quantitative polymerase chain reaction (qPCR) technique was used to analyze mRNA expression profiles involving ferroptosis, cell proliferation, and cell death (total 69 genes) in tumor tissues of the **15**-treated and control groups (Figure 6, top and bottom panels).

**Figure 7:**
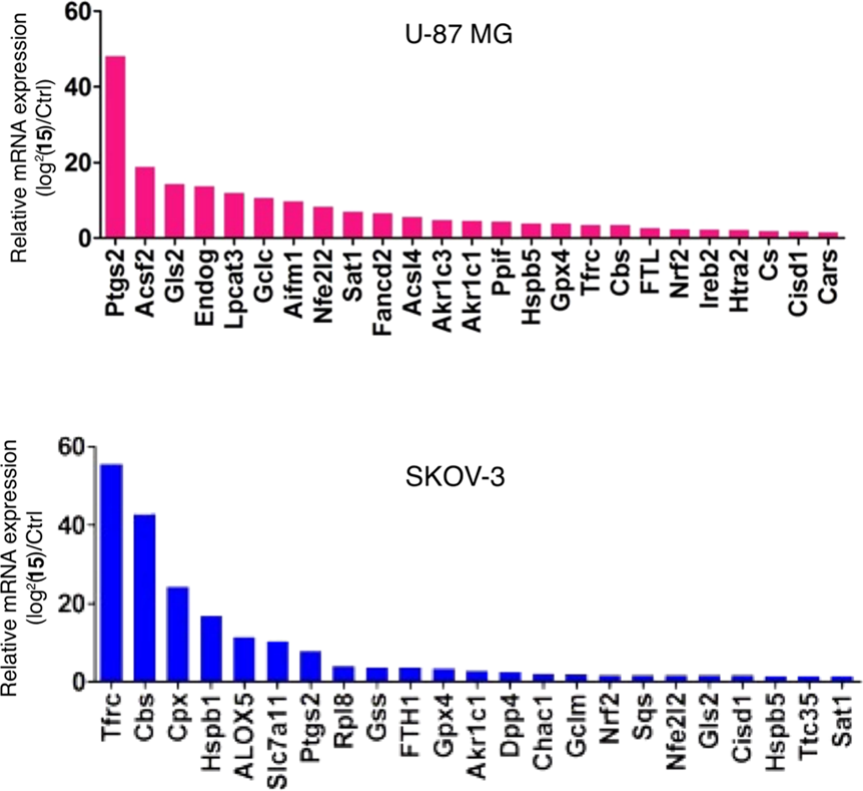
Expression levels of genes that showed ≥1.5 significance.

**Figure 8:**
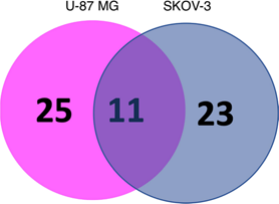
Venn diagram showing the overlapping 11 genes between the U-87 MG and SKOV-3 tumors.

According to the 2^–ΔΔCt^ method,^27^ significance was calculated as follows:
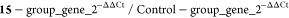


A value ≥1.5 was considered significant for indicating an increased expression level. Accordingly, the expression levels of 25 genes in U-87 MG tumor tissues derived from the **15**-treated group were significantly elevated compared to those in the control group (Figure 7, top panel). The expression levels of 23 genes in SKOV-3 tumor tissues derived from the **15**-treated group were significantly elevated compared to the control group (Figure 7, bottom panel). Eleven genes (*Ptgs2*, *Gls2*, *Nfe2l2*, *Sat1*, *Akr1c1*, *Hspb5*, *Gpx4*, *Tfrc*, *Cbs*, *Nrf2*, and *Cisd1*) were present in both groups (Figure 8).

**Figure 9:**
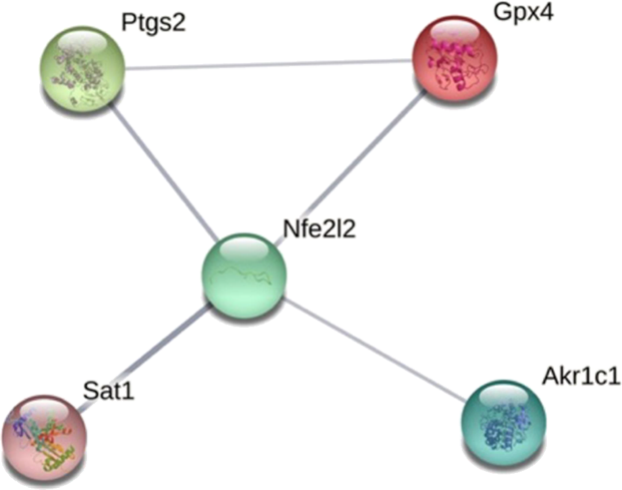
Protein interaction network of **15** by STRING v11.

**Figure 10:**
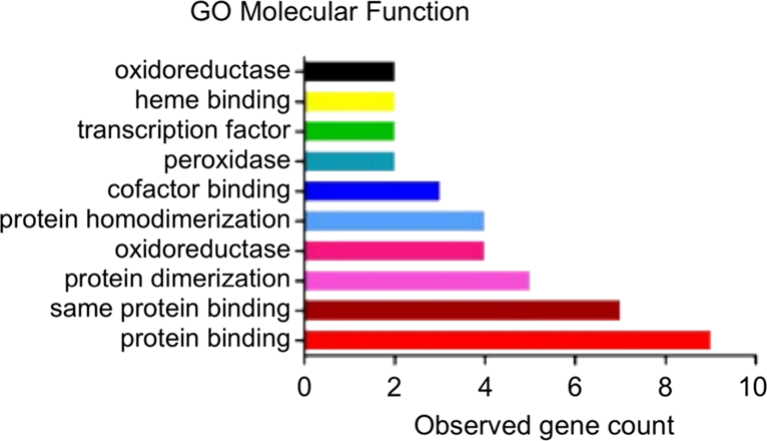
GO analysis of molecular functions of **15**.

Using the protein–protein interaction prediction tool STRING v11,^28,29^ we analyzed the protein network of the above 11 genes related to ferroptosis. Among them, the results suggested that the *Ptgs2*/*Nfe2l2*/*Sat1*/*Akr1c1*/*Gpx4* genes have potential to regulate protein–protein interactions (Figure 9): the *Ptgs2* gene can cause angiogenesis, differentiation, and promotion of cancer through dysregulation of COX-2;^30^ the *Nfe2l2* gene encodes for NRF2 that in humans is a central regulator of redox, metabolic, and protein homeostasis;^31^ the *Sat1* gene encodes for an acetyltransferase that is involved in the regulation of the intracellular concentration of polyamines and their transport out of cells;^32^ the *Akr1c1* gene encodes for the AKR1C1 enzyme that catalyzes the reduction of aldehydes and ketones to their corresponding alcohol and is over-expressed in the lungs, ovary, uterine cervix, skin, and colon carcinomas;^33^ the *GPX4* gene encodes for the enzyme GPX4, which protects cells against membrane lipid peroxidation. GPX4 is an essential regulator of ferroptotic cancer cell death.^34^ A graph from Gene Ontology (GO) analysis^35^ (Figure 10) shows that **15** could affect molecular functions, such as protein binding, protein dimerization activity, and oxidoreductase activity.

**Figure 11:**
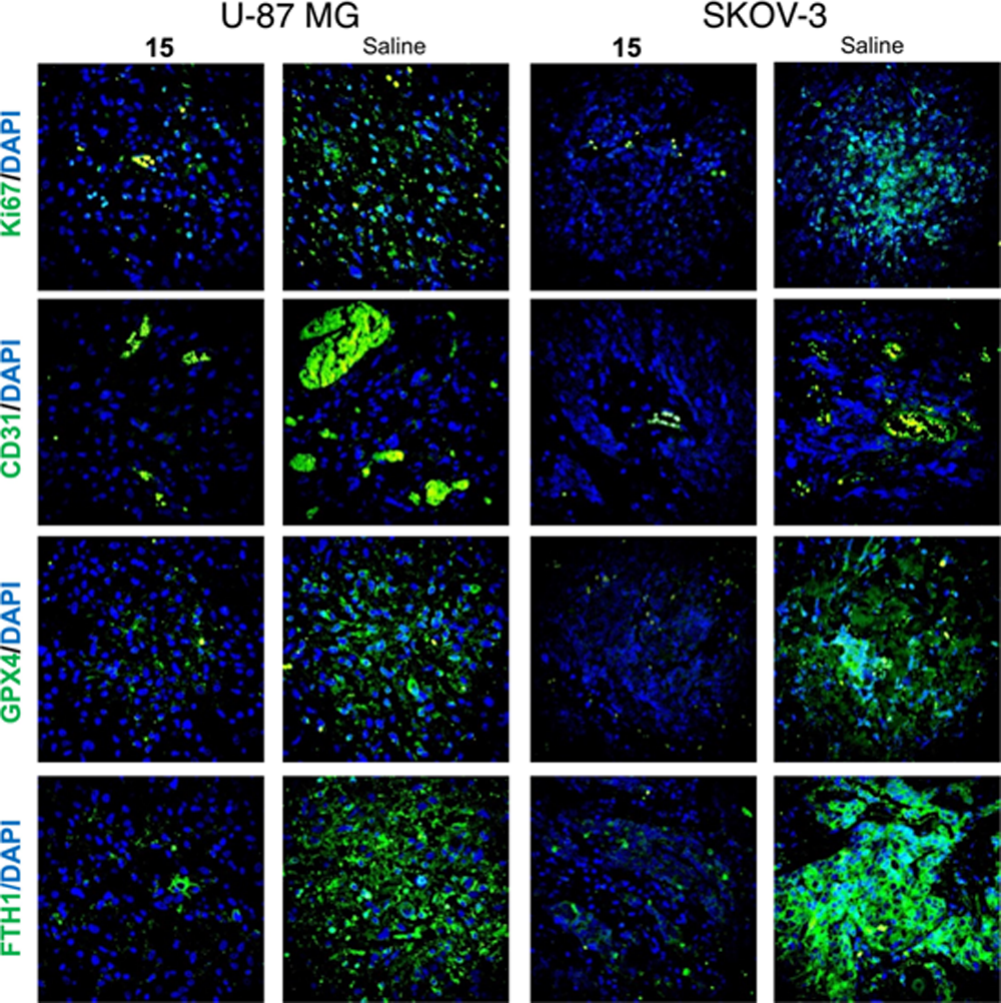
Compound **15** regulated the expression of proteins involved in ferroptosis. Magnification 200×.

Immunofluorescence staining showed that the tumor tissues derived from the **15**-treated groups have significantly lower expression of Ki67 and CD31 and of ferroptosis negative regulation proteins GPX4 and FTH1. The proliferation marker Ki67 is a nuclear protein that is strongly associated with tumor cell proliferation and is an established prognostic indicator for cancer assessment by biopsy.^36^ CD31 is a well-defined marker of angiogenesis, along with the vascular endothelial growth factor (VEGF).^37^ CD31 is highly expressed on the surface of endothelial cells, and CD31 is involved in angiogenesis in early breast cancer.^38^ GPX4 and FTH1 are ferroptosis negative regulation proteins. The selenoenzyme GPX4 reduces membrane phospholipid hydroperoxides and maintains cellular redox homeostasis, using glutathione (GSH) as a cofactor.^39^ Inactivation or depletion of GPX4 in a variety of cell types can induce ferroptosis.^34^ FTH1 has ferroxidase activity, which specifically oxidizes ferrous iron (FeII) to ferric iron (FeIII). FTH1 regulates angiogenesis during inflammation and malignancy, interacts with several signaling elements involved in critical cellular pathways, and activates p53 under oxidative stress. FTH1 acts as a tumor suppressor in nonsmall cell lungs and breast and ovarian cancers, as well as as a tumor promoter in metastatic melanoma cells.^40^ In summary, we conclude that **15** promoted ferroptosis in tumor tissues by stimulating the ferroptosis protein regulation network (Figure 11).

#### Metabolic Stability

Compound **15** was assessed for its metabolic stability to phase I oxidative metabolism using mouse and human liver microsomes (Table 2), with 7-ethoxycoumarin (7-EC) and propranolol as control compounds. Compound **15** was highly metabolized after incubation with mouse liver microsomes, showing a very high intrinsic clearance value of 461 μL/min/mg protein. However, compound **15** was found to be much more metabolically stable when incubated with human liver microsomes showing a medium intrinsic clearance of 36 μL/min/mg protein (Table 3). Liquid chromatography tandem mass spectroscopy (LC–MS/MS) analyses were carried out using an ESI(+) interface in multiple reaction monitoring (MRM) mode. Conditions and MRM transitions applied to the compounds are described in Table 4. The metabolic stability profile of **15** did not differ much from propranolol. From a drug development point of view, the stability shown in the presence of human liver microsome enzymes may represent a good starting point for compound optimization.

**Table 2:** In Vitro Determination of the Metabolic Stability after Incubation with Mouse and Human Liver Microsomes^a^

compd	human liver microsomes (HLM)		mouse liver microsomes (MLM)	
	μL/min/mg protein	min	μL/min/mg protein	min
	Cli ± SD	*t*_1/2_ ± SD	Cli ± SD	*t*_1/2_ ± SD
**15**	35.9 ± 0.2	38.7 ± 0.2	461.1 ± 45.5	3.0 ± 0.3
7-EC^b^	231.3 ± 37.5	6.1 ± 1.0	710.8 ± 1.2	2.0 ± 0.1
Pro.^c^	45.9 ± 2.7	30.2 ± 1.8	235.1 ± 24.0	5.9 ± 0.6

a Results are expressed as the mean ± SD, *n* = 2.

b 7-EC, ethoxycoumarin.

c Pro., propranolol. The standard compounds 7-EC and Prop. showed metabolic stability in agreement with the literature and internal validation data.

**Table 3:** In Vitro Clearance Classification^a^

classification	Cli (μL/min/mg)		
	low Cli	medium Cli	high Cli
mouse	≤2.5	2.5–66	>66
human	≤1.8	1.8–48	>48

a Data obtained from refs (41−43).

**Table 4:** Compound MRM Transitions and Conditions

compound	parent ion	product ion	DP (V)	CE (eV)
7-EC	190.9	163.0	56	23
propranolol	260.4	183.2	40	25
verapamil	455.4	165.1	31	35
**15**	406.1	238.3	46	35

#### Druglike Properties

The ADME profile of **15** was predicted through representative descriptors by the SwissADME web site^44^ (Table 5). According to the metabolic stability data reported in Table 2, compound **15** was predicted to be the substrate of the highly expressed CYP450 2C9, 2D6, and 3A4 isoforms.^45^ The compound does not violate the Lipinski^46^ and Veber^47^ rules, may have good absorption after oral administration, and shows low likelihood of in vivo toxicological outcome (3/75 rule).^48^

**Table 5:** Compound **15** ADME Profile

comp	log*P*^a^	MW^b^	logSw^c^	tPSA^d^	GI^e^	P-gp^f^	Lipinski^g^	Veber^h^	3/75^i^
**15**	3.98	525.02	–4.91	75.47	High	No	0	0	Low

a Logarithm of the partition coefficient between *n*-octanol and water computed by the XLOGP3 method.^49^

b Molecular weight.

c LogSw represents the logarithm of compound water solubility computed by the ESOL method. LogSw predicted compound aqueous solubility values: >−10: insoluble, >−6: poorly soluble, >−4: moderately soluble, >−2: soluble, >0: high soluble.^50^

d Molecular polar surface area, this parameter has been shown to correlate with human intestinal absorption (<140).^51^

e GI Gastrointestinal absorption boiled egg method.^52^

f P-gp P-glycoprotein substrate SVM model.^53^

g Violation of the rule of five (MW < 500; log*P* < 5; HBD ≤ 10; HBA ≤ 5).^46^

h Veber’s rule matching.^47^

i 3/75 rule matching (log*P* > 3 and topological PSA < 75 Å^2^).^48^

## Conclusions

We synthesized new aroyl diheterocyclic pyrrole (ARDHEP) derivatives as inhibitors of tubulin polymerization with a strong interaction with the colchicine site. Compound **15** exhibited the hallmarks of ferroptosis and inhibited the cell viability of human GBM U-87 MG and OC OVCAR-3 cells with IC_50_ values of 10.06 and 2.85 nM, respectively. In OVCAR-3 cells, **15** induced an increase of cleaved PARP, starting from 5 nM, while the proliferation of GBM cells U-87 MG was accompanied by the PARP cleavage starting from 20 nM. At 0.1 μM, **15** was not toxic for normal human primary T lymphocytes. In the tumor tissues of mice treated with **15**, the levels of LPO, MDA, and lactic acid were appreciably higher, the levels of T-GSH and ATP were significantly lower and the concentrations of Fe^2+^ were higher as compared to the control groups. These results suggested that in vivo inhibition of cancer cell proliferation by **15** went through stimulation of oxidative stress injury and Fe^2+^ accumulation. qPCR analysis of the mRNA expression profiles involving ferroptosis, cell proliferation, and cell death (total 69 genes) showed significant expression levels of 25 genes in U-87 MG and 23 genes in SKOV-3 tumor tissues derived from the **15**-treated groups. Eleven genes (*Ptgs2*, *Gls2*, *Nfe2l2*, *Sat1*, *Akr1c1*, *Hspb5*, *Gpx4*, *Tfrc*, *Cbs*, *Nrf2*, and *Cisd1*) were present in both groups. Analysis of genes correlated with ferroptosis showed that the *Ptgs2*/*Nfe2l2*/*Sat1*/*Akr1c1*/*Gpx4* genes have potential to regulate protein–protein interactions. According to the GO analysis, compound **15** could affect molecular functions, such as protein binding, protein dimerization activity, and oxidoreductase activity. Immunofluorescence staining showed that the tumor tissues derived from the **15**-treated groups have significantly lower expressions of Ki67 and CD31 and of ferroptosis negative regulation proteins GPX4 and FTH1. Dimeric tubulin is associated with mitochondria^54^ with high affinity through the mitochondrial outer membrane where the voltage-dependent anion channel (VDAC) is the most abundant protein.^55,56^ The association of tubulin with VDAC induces highly voltage-sensitive reversible blockage of the single channel and hampers the cellular metabolism.^57^ The microtubule-targeting agents, by binding directly to the tubulin associated with VDAC, cause depolarization of the mitochondrial membrane and interferes with mitochondrial function.^58,59^ By interaction with the VDAC, the ferroptosis inducer erastin causes ferroptotic (nonapoptotic) cell death and abnormal functioning of mitochondria.^60,61^ We hypothesize that the novel tubulin polymerization inhibitor **15** may interact with tubulin-associated-VDAC, thereby interfering with the function of the mitochondrial activity. The interaction between tubulin and VDAC has been reported as novel target for inducing ferroptosis in cancer cells.^62^ Compound **15** was metabolically stable when incubated with human liver microsomes and showed a medium intrinsic clearance of 36 μL/min/mg protein. In summary, we described the synthesis and antitumor activities in vitro and in vivo of a tubulin polymerization inhibitor, compound **15**, that induced cell death and presented the typical hallmarks of ferroptosis rather than conventional apoptosis. The biological profile of **15**, together with its stability in the presence of human liver microsome enzymes, highlights compound **15** as a robust lead compound for further optimization to provide new anticancer drugs based on alternative mechanisms of action.

## Experimental Section

### Chemistry

All reagents and solvents were handled according to the material safety data sheet of the supplier and were used as purchased without further purification. MW-assisted reactions were performed on a CEM Discover SP single-mode reactor equipped with an Explorer 72 autosampler, controlling the instrument settings by PC-running CEM Synergy 1.60 software. Closed vessel experiments were carried out in capped MW-dedicated vials (10 mL) with a cylindrical stirring bar (length 8 mm, diameter 3 mm). Stirring, temperature, irradiation power, maximum pressure (Pmax), pressure set point, times at set point, delta pressure, PowerMAX (simultaneous cooling-while-heating), ActiVent (simultaneous venting-while-heating), and ramp and hold times were set as indicated. Reaction temperature was monitored by an external CEM fiber optic temperature sensor. After completion of the reaction, the mixture was cooled to 25 °C via air-jet cooling. Organic solutions were dried over anhydrous sodium sulfate. Evaporation of solvents was carried out on a Büchi Rotavapor R-210 equipped with a Büchi V-850 vacuum controller and a Büchi V-700 vacuum pump. Column chromatography was performed on columns packed with silica gel from Macherey-Nagel (70–230 mesh). Silica gel thin layer chromatography (TLC) cards from Macherey-Nagel (silica gel-precoated aluminum cards with fluorescent indicator visualizable at 254 nm) were used for TLC. Developed plates were visualized with a Spectroline ENF 260C/FE UV apparatus. Melting points (m.p.) were determined on a Stuart Scientific SMP1 apparatus and are uncorrected. Infrared (IR) spectra were recorded on a PerkinElmer Spectrum 100 Fourier transform-IR (FT-IR) spectrophotometer equipped with a universal attenuated total reflectance accessory and IR data acquired and processed by PerkinElmer Spectrum 10.03.00.0069 software. Band positions and absorption ranges are given in cm^–1^. Proton nuclear magnetic resonance (1H NMR) spectra were recorded with a Bruker Avance (400 MHz) spectrometer in the indicated solvent, and the corresponding fid files were processed by MestreLab Research SL MestreReNova 6.2.1–769 software. Chemical shifts are expressed in δ units (ppm) from tetramethylsilane. Compound purity was checked by high-pressure liquid chromatography (HPLC). Purity of tested compounds was found to be >95%. The HPLC system used (Thermo Fisher Scientific Inc. Dionex UltiMate 3000) consisted of an SR-3000 solvent rack, an LPG-3400SD quaternary analytical pump, a TCC-3000SD column compartment, a DAD-3000 diode array detector, and an analytical manual injection valve with a 20 μL loop. Samples were dissolved in acetonitrile (1 mg/mL). HPLC analysis was performed by using a Thermo Fisher Scientific Inc. An Acclaim 120 C18 column (5 μm, 4.6 mm × 250 mm) at 25 ± 1 °C, with an appropriate solvent gradient (acetonitrile/water), flow rate of 1.0 mL/min, and a signal detector at 206, 230, 254, and 365 nm were used. Chromatographic data were acquired and processed by Thermo Fisher Scientific Inc. Chromeleon 6.80 SR15 Build 4656 software. Ultrahigh-performance liquid chromatography (UHPLC) experiments were carried out on an Accela UHPLC System Thermo Fisher Scientific (San Jose, CA), which consisted of an Accela 1250 Pump, an Accela autosampler, and an Accela PDA photodiode array detector. Chromatographic data were collected and processed using the Thermo Xcalibur Chromatography Manager software, version 1.0. A guard cartridge system (SecurityGuard Ultra UHPLC) has been connected to an analytical column Kinetex 2.6 μm EVO C18 100 Å 100 × 3.0 mm (L × I.D.), both from Phenomenex, Torrance, CA, USA. All analyses were performed at 30 °C, and the mobile phase was filtered through 0.2 μm Omnipore filters (Merck Millipore, Darmstadt, Germany). The mobile phase was delivered at a total flow rate of 0.5 mL/min. The analyses were carried out in elution gradient. Specific mobile phase and gradient are reported for each compound in Figures S3–S17, Supporting Information. Relative areas (%) recorded at 254 nm are shown in Table S1, Supporting Information. Analyses were performed in triplicate. Acetonitrile, methanol, water, and trifluoroacetic acid of HPLC gradient grade were purchased from Sigma Aldrich (St. Louis, MO).

### Preparation of Compounds **2–5**, **13**, and **14**

#### (1-(Pyridin-2-yl)-1*H*-pyrrol-3-yl)(3,4,5-trimethoxyphenyl)methanone (**2**)

A mixture of **17** (77 mg, 0.296 mmol) (24), 2-iodopyridine (94 mg, 0.457 mmol), copper(I) iodide (28 mg, 0.145 mmol), cesium carbonate (88 mg, 0.457 mmol), and 1,10-phenanthroline (5 mg, 0.030 mmol) in 1,4-dioxane (2 mL) was placed into a microwave cavity (closed vessel mode, *P*_max_ = 250 psi). A starting MW irradiation of 300 W was used, the temperature being ramped from 25 to 200 °C. Once 200 °C was reached, taking about 5 min, the reaction mixture was held at this temperature for 25 min. The reaction mixture was diluted with water and extracted with ethyl acetate. The organic layer was washed with brine, dried, and filtered. Evaporation of the solvent gave a residue that was purified by column chromatography (silica gel, *n*-hexane:ethyl acetate = 1:1) to furnish **2** (79 mg, 79%), m.p. 120–125 °C (from ethanol). ^1^H NMR (DMSO-*d*_6_): δ 3.75 (s, 3H), 3.84 (s, 6H), 6.79–6.80 (m, 1H), 7.11 (s, 2H), 7.33–7.36 (m, 1H), 7.83–7.84 (m, 1H), 7.92–7.97 (m, 2H), 8.28–8.29 (m, 1H), 8.48 ppm (d, *J* = 4.4 Hz, 1H). IR: ν 1642 and 3118 cm^–1^.

#### (1-(Pyridin-3-yl)-1*H*-pyrrol-3-yl)(3,4,5-trimethoxyphenyl)methanone (**3**)

The compound was synthesized as **2** starting from **17** (24) and 3-iodopyridine. Yield 80%, m.p. 115–116 °C (from ethanol). ^1^H NMR (DMSO-*d*_6_): δ 3.74 (s, 3H), 3.85 (s, 6H), 6.81–6.84 (m, 1H), 7.12 (s, 2H), 7.52–7.55 (m, 1H), 7.62–7.65 (m, 1H), 8.15–8.18 (m, 2H), 8.54 (d, *J* = 4.5 Hz, 1H), 8.98–9.01 (m, 1H) ppm. IR: ν 1581 and 3114 cm^–1^.

#### (1-(Pyridin-4-yl)-1*H*-pyrrol-3-yl)(3,4,5-trimethoxyphenyl)methanone (**4**)

The compound was synthesized as **2** starting from **17** (24) and 4-iodopyridine. Yield 84%, m.p. 134–137 °C (from ethanol). ^1^H NMR (DMSO-*d*_6_): δ 3.74 (s, 3H), 3.84 (s, 6H), 6.82–6.85 (m, 1H), 7.11 (s, 2H), 7.77–7.78 (m, 1H), 7.85–7.86 (m, 2H), 8.27–8.28 (m, 1H), 8.63–8.65 ppm (m, 2H). IR: ν 1603 and 3111 cm^–1^.

#### (1-(Pyrimidin-2-yl)-1*H*-pyrrol-3-yl)(3,4,5-trimethoxyphenyl)methanone (**5**)

The compound was synthesized as **2** starting from **17** (24) and 2-bromopyrimidine. Yield 70%, m.p. 180–183 °C (from ethanol). ^1^H NMR (DMSO-*d*_6_): δ 3.62 (s, 3H), 3.71 (s, 6H), 6.70–6.73 (m, 1H), 7.00 (s, 2H), 7.35 (t, *J* = 4.8 Hz, 1H), 7.72–7.75 (m, 1H), 8.10–8.11 (m, 1H), 8.74 ppm (d, *J* = 4.8 Hz, 2H). IR: ν 1574 and 2836 cm^–1^.

#### (4-Phenyl-1-(pyrimidin-2-yl)-1*H*-pyrrol-3-yl)(3,4,5-trimethoxyphenyl)methanone (**13**)

The compound was synthesized as **2** starting from **18** (23) and 2-bromopyrimidine. Yield 23%, m.p. 128–132 °C (from ethanol). ^1^H NMR (DMSO-*d*_6_): δ 3.71 (s, 3H), 3.74 (s, 6H), 7.09 (s, 2H), 7.22–7.31 (m, 3H), 7.37–7.40 (m, 2H), 7.45–7.50 (m, 1H), 7.97–7.99 (m, 1H), 8.13–8.14 (m, 1H), 8.85–8.88 ppm (m, 2H). IR: ν 1033 and 2936 cm^–1^.

#### (4-Phenyl-1-(pyrimidin-5-yl)-1*H*-pyrrol-3-yl)(3,4,5-trimethoxyphenyl)methanone (**14**)

The compound was synthesized as **2** starting from **18** (23) and 5-bromopyrimidine. Yield 60%, m.p. 167–171 °C (from ethanol). ^1^H NMR (DMSO-*d*_6_): δ 3.71 (s, 3H), 3.77 (s, 6H), 7.13 (s, 2H), 7.19–7.24 (m, 3H), 7.29 (d, *J* = 7.6, 2H), 7.36–7.38 (m, 2H), 7.92 (d, *J* = 2.3, 1H), 8.17 (d, *J* = 2.3, 1H), 9.13–9.14 (m, 1H), 9.31–9.32 ppm (m, 2H). IR: ν 1033 and 2923 cm^–1^.

### Preparation of Compounds **6**, **9–12**, **15**, and **16**

#### (4-Phenyl-1-(pyridin-2-yl)-1*H*-pyrrol-3-yl)(3,4,5-trimethoxyphenyl)methanone (**6**)

A mixture of **18** (206 mg, 0.611 mmol) (23), 2-bromothiazole (160 mg, 0.780 mmol), copper(I) iodide (52 mg, 0.300 mmol), cesium carbonate (297 mg, 0.912 mmol) and 1,10-phenanthroline (9 mg, 0.052 mmol) in 1,4-dioxane (4 mL) was stirred at 100 °C for 24 h under an argon stream. After cooling, the reaction mixture was diluted with water and extracted with ethyl acetate. The organic layer was washed with brine, dried and filtered. Evaporation of the solvent gave a residue that was purified by silica gel column chromatography (silica gel, petroleum ether:ethyl acetate = 8:2) to furnish **6** (251 mg, 99%), m.p. 150–152 °C (from CH_2_Cl_2_/*n*-hexane). ^1^H NMR (DMSO-*d*_6_): δ 3.69 (s, 3H), 3.74 (s, 6H), 7.12 (s, 2H), 7.30–7.36 (m, 5H), 7.85–7.91 (m, 2H), 7.99–8.00 (m, 1H), 8.18–8.21 (m, 1H), 8.65–8.66 ppm (m, 2H). IR ν 1228 and 2834 cm^–1^.

#### (4-(Furan-2-yl)-1-(pyridin-2-yl)-1*H*-pyrrol-3-yl)(3,4,5-trimethoxyphenyl)methanone (**9**)

The compound was synthesized as **6** starting from **19** (23) and 2-bromopyridine. Yield 51%, m.p. 129–131 °C (from ethanol). ^1^H NMR (CDCl_3_): δ 3.88 (s, 6H), 3.94 (s, 3H), 6.42–6.43 (m, 1H), 6.95 (d, *J* = 3.3 Hz, 1H), 7.17 (s, 2H), 7.20–7.27 (m, 1H), 7.37–7.44 (m, 2H), 7.82 (t, *J* = 7.5 Hz, 1H), 7.88–7.89 (m, 1H), 8.00–8.01 (m, 1H), 8.46 ppm (d, *J* = 4.8 Hz, 1H). IR: ν 1121 and 3136 cm^–1^.

#### (4-(Furan-2-yl)-1-(pyridin-3-yl)-1*H*-pyrrol-3-yl)(3,4,5-trimethoxyphenyl)methanone (**10**)

The compound was synthesized as **6** starting from **19** (23) and 3-iodopyridine. Yield 41%, m.p. 124–128 °C (from ethanol). ^1^H NMR (CDCl_3_): δ 3.87 (s, 6H), 3.92 (s, 3H), 6.41–6.42 (m, 1H), 6.89 (d, *J* = 3.6 Hz, 1H), 7.15 (s, 2H), 7.35–7.36 (m, 1H), 7.44–7.52 (m, 3H), 7.76–7.79 (m, 1H), 8.61–8.62 (m, 1H), 8.81–8.82 ppm (m, 1H). IR: ν 1127 and 2938 cm^–1^.

#### (4-(Furan-2-yl)-1-(pyridin-4-yl)-1*H*-pyrrol-3-yl)(3,4,5-trimethoxyphenyl)methanone (**11**)

The compound was synthesized as **6** starting from **19** (23) and 4-iodopyridine. Yield 30%, m.p. 155–158 °C (from ethanol). ^1^H NMR (CDCl_3_): δ 3.87 (s, 6H), 3.93 (s, 3H), 6.41–6.42 (m, 1H), 6.89 (d, *J* = 3.6 Hz, 1H), 7.15 (s, 2H), 7.35–7.36 (m, 1H), 7.44–7.52 (m, 3H), 7.76–7.79 (m, 1H), 8.61–8.62 (m, 1H), 8.81–8.82 ppm (m, 1H). IR: ν 1127 and 2938 cm^–1^.

#### (4-(Furan-2-yl)-1-(pyrazin-2-yl)-1*H*-pyrrol-3-yl)(3,4,5-trimethoxyphenyl)methanone (**12**)

The compound was synthesized as **6** starting from **19** and 2-iodopyrazine. Yield 57%, m.p. 174–176 °C (from ethanol). ^1^H NMR (CDCl_3_): δ 3.88 (s, 6H), 3.94 (s, 3H), 6.41–6.42 (m, 1H), 6.92 (d, *J* = 3.0 Hz, 1H), 7.17 (s, 2H), 7.36–7.37 (m, 1H), 7.90 (d, *J* = 2.4 Hz, 1H), 7.99 (d, *J* = 2.4 Hz, 1H), 8.42–8.43 (m, 1H), 8.50–8.51 (m, 1H), 8.86–8.87 ppm (m, 1H). IR: ν 1122 and 2939 cm^–1^.

#### (4-(Furan-2-yl)-1-(pyrimidyl-2-yl)-1*H*-pyrrol-3-yl)(3,4,5-trimethoxyphenyl)methanone (**15**)

The compound was synthesized as **6** starting from **19** and 2-bromopyrimidine. Yield 8%, m.p. 197–199 °C (from ethanol). ^1^H NMR (CDCl_3_): δ 3.89 (s, 3H), 3.94 (s, 6H), 6.42 (s, 1H), 6.95 (d, *J* = 3.0 Hz, 1H), 7.16–7.19 (m, 2H), 7.39 (s, 1H), 8.20 (s, 2H), 8.67 (d, *J* = 4.8 Hz, 2H). IR: ν 1033 and 2936 cm^–1^.

#### (4-(Furan-2-yl)-1-(pyrimidin-5-yl)-1*H*-pyrrol-3-yl)(3,4,5-trimethoxyphenyl)methanone (**16**)

The compound was synthesized as **6** starting from **19** and 5-iodopyrimidine. Yield 24%, m.p. 184–185 °C (from ethanol). ^1^H NMR (CDCl_3_): δ 3.87 (s, 6H), 3.93 (s, 3H), 6.41–6.42 (m, 1H), 6.85 (d, *J* = 3.3 Hz, 1H), 7.14 (s, 2H), 7.36–7.37 (m, 1H), 7.48 (d, *J* = 2.4 Hz, 1H), 7.53 (d, *J* = 2.4 Hz, 1H), 8.93–8.94 (m, 2H), 9.21–9.22 ppm (m, 1H). IR: ν 1122 and 2939 cm^–1^.

### Preparation of Compounds **7** and **8**

#### (4-Phenyl-1-(pyridin-3-yl)-1*H*-pyrrol-3-yl)(3,4,5-trimethoxyphenyl)methanone (**7**)

A solution of **18** (200 mg, 0.593 mmol) (23), pyridin-3-ylboronic acid (103 mg, 0.840 mmol), copper(II) acetate (108 mg, 0.59 mmol), and triethylamine (79 mg, 0.109 mL, 0.780 mmol) in 1,2-dichloroethane (5 mL) was stirred at 40 °C for 18 h under an argon steam. After cooling, the reaction mixture was diluted with water and extracted with ethyl acetate; the organic layer was washed with brine, dried, and filtered. Evaporation of the solvent gave a residue that was purified by column chromatography (silica gel, ethyl acetate–petroleum ether = 7:3) to furnish **7** (0.030 g, 12%), m.p. 171–173 °C (from ethanol). ^1^H NMR (CDCl_3_): δ 3.79 (s, 6H), 3.86 (s, 3H), 7.10 (s, 2H), 7.20–7.22 (m, 2H), 7.24–7.32 (m, 3H), 7.86–7.88 (m, 2H), 7.99–8.05 (m, 1H), 8.25–8.34 (m, 1H), 8.80–8.84 ppm (m, 2H). IR: ν 1325 and 2926 cm^–1^.

#### (4-Phenyl-1-(pyridin-4-yl)-1*H*-pyrrol-3-yl)(3,4,5-trimethoxyphenyl)methanone (**8**)

The compound was synthesized as **7** starting from **18** (23) and pyridin-4-ylboronic acid. Yield 9%, m.p. 158–162 °C (from ethanol). ^1^H NMR (DMSO-*d*_6_): δ 3.71 (s, 3H), 3.76 (s, 6H), 7.12 (s, 2H), 7.18–7.25 (m, 2H), 7.28–7.38 (m, 3H), 7.88–7.90 (m, 2H), 7.99–8.00 (m, 1H), 8.19–8.20 (m, 1H), 8.62–8.63 ppm (m, 2H). IR: ν 1122 and 2924 cm^–1^.

### Biology

#### Tubulin Assembly and Colchicine Binding Assays

The assembly reaction mixtures contained 0.8 M monosodium glutamate (pH 6.6 with HCl in a 2 M stock solution), 10 μM tubulin, 4% (v/v) DMSO, and varying concentrations of the drug. Following a 15 min preincubation at 30 °C, samples were chilled on ice, GTP to 0.4 mM was added, and turbidity development was followed at 350 nm in a temperature-controlled recording spectrophotometer for 20 min at 30 °C. The extent of reaction was measured. Full experimental details were previously described.^63^ For the colchicine binding assay, reaction mixtures contained 1.0 μM tubulin, 5.0 μM [^3^H]colchicine, and 5.0 or 1.0 μM inhibitor and were incubated for 10 min at 37 °C. Complete details were described previously.^64^

#### Cell Cultures

Cell lines were obtained from the American Type Culture Collection (ATCC), Rockville, MD. U87 MG cells were cultured in minimum essential medium (MEM) supplemented with 10% fetal bovine serum, 1% penicillin/streptomycin, 1% glutamine, 1% sodium pyruvate, and 1% nonessential amino acids. OVCAR-3 cells were cultured in Dulbecco’s modified eagle medium (DMEM) purchased from Sigma Aldrich (cat. no. D6546) supplemented with 20% fetal bovine serum,1% penicillin/streptomycin, 1% glutamine, 1% sodium pyruvate, 1% Hepes, and 0.01 μg/mL insulin (Sigma Aldrich, cat. no. 19278). SKOV-3 cells, with invasive capacity superior to the OVCAR-3,^65^ were cultured in Roswell Park Memorial Institute medium (RPMI) media, supplemented with 10% fetal bovine serum, 100 U/mL penicillin and streptomycin, and 2 mmol/L glutamine. Healthy donors’ peripheral blood mononuclear cells (PBMCs) were isolated by Lymphoprep (Nycomed) gradient centrifugation. T lymphocytes were negatively selected from PBMCs using a magnetic Dynabeads Untouched Human T Cells Kit (Thermo Fisher Scientific) following the manufacturer’s instructions.

#### Cell Viability Assays

The methodology for the evaluation of the growth of human MCF-7 breast carcinoma cells was previously described, except that cells were grown for 96 h for IC_50_ determinations.^66^ U-87 MG and OVCAR-3 proliferation assays were performed plating 10^4^ cells/cm^2^ in a Multiwell 24. The day after, cells were treated with compound **15**, and DMSO was used as a control. Cells were counted 48 h later with a Bürker counting chamber, after dilution in TrypanBlue (#T6146 Sigma-Aldrich). IC_50_ was calculated by using data from the dose–response curves after 48 h of drug treatment using Graphpad Prism 7.0 software, as previously described.^67^

Apoptotic cell death was evaluated using the APC Annexin-V Apoptosis Detection Kit with PI (Thermo Fisher Scientific). Briefly, 1.5 × 10^6^/mL cells were cultured in 48-well plates, untreated or treated with different concentrations of **15** for 48 or 72 h. Cells were then stained using annexin-V/APC and propidium iodide according to the manufacturer’s instructions. Cell populations were acquired using a FACS Canto II flow cytometer (BD Biosciences). Flow cytometric analysis was performed using Flow Jo Flow Cytometric Analysis Software.

#### Western Blotting

Western blotting was performed as previously described^68^ by lysing cells in denaturing buffer sodium dodecyl sulfate (SDS)–urea. Extracts were sonicated, their protein quantified, and aliquots were loaded into an SDS–polyacrylamide gel. After electrophoresis, the proteins in the gel were transferred onto a nitrocellulose membrane (cat. no. NBA085C001EA, PerkinElmer), which then was blocked with 5% milk in TBS-T (Tris HCl with 0.1% Tween 20) and incubated with primary antibodies overnight: PARP antibody diluted 1:1000 (cat. no. 9542S CST) and actin antibody diluted 1:10,000 (cat. no. A5441, Sigma Aldrich). The next day, the membrane was extensively washed with TBS-T and incubated with horse radish peroxidase (HRP)-conjugated secondary antibodies diluted in the 5% milk solution. Detection of the HRP signal was performed using ECL (cat. no. K-12045-D50, Advansta).

#### Statistical Analysis

All experiments were performed multiple times to reach statistical significance, as specified in the figure legends. Statistical analysis was performed using GraphPad Prism version 7.0 for Mac. IC_50_ values were calculated by using a nonlinear regression formula, using the percentage of cells number and the logarithmic value of drug concentrations. Data were analyzed with analysis of variance (one-way ANOVA test). Data with *p < 0.05 were considered statistically significant.

#### In Vivo Xenograft Experiments

Briefly, all 10 week-old female BALB/C^nu/nu^ mice (24 mice) were purchased from the Shanghai University of Traditional Chinese Medicine with Institutional Animal Care and Use Committee approval in accordance with institutional guidelines. All mice were randomly divided into four groups. In #1 group (4 mice), 1 × 10^8^ cells/mL from U-87 MG at the logarithmic growth phase were harvested and inoculated subcutaneously, followed by intraperitoneal injection of 100 μL of **15** (25 mg/kg) every 2 days. In #2 group (4 mice), 1 × 10^8^ cells/mL from U-87 MG at the logarithmic growth phase were harvested and inoculated subcutaneously, followed by intraperitoneal injection of 100 μL of saline every 2 days. In #3 group (4 mice), 1 × 10^8^ cells/mL from SKOV-3 at the logarithmic growth phase were harvested and inoculated subcutaneously, followed by intraperitoneal injection of 100 μL **15** (25 mg/kg) every 2 days. In #4 group (4 mice), 1 × 10^8^ cells/mL from SKOV-3 at the logarithmic growth phase were harvested and inoculated subcutaneously, followed by intraperitoneal injection of 100 μL of saline every 2 days. After continuous feeding for 40 days, the mice were sacrificed, and the tumors were removed. The tumors were weighed, and the volumes were calculated using the following formula: Tumor volume (cm^3^) = (*ab*^2^)/2 (*a*: the longest axis (cm), *b*: the shortest axis (cm)).

#### HE Staining

Tissue samples were fixed in 4% paraformaldehyde, dehydrated, and embedded in paraffin. The paraffin-embedded tissues were cut into 4 μm sections using a microtome, and the sections were affixed onto glass slides. Subsequently, the sections were dewaxed using xylene and subjected to dehydration in an ethanol gradient. The sections were stained with hematoxylin (H) for 5 min at room temperature, and then, 1% ethanol was added for 30 s for differentiation. Afterward, aqueous ammonia was added for 1 min for blueing, followed by rinsing in distilled water for 5 min. Subsequently, the sections were stained with eosin (E) for 2 min at room temperature and then rinsed with distilled water for 2 min. Then, decolorization over an ethanol gradient was performed, and xylene was added for 2 min for clearing. Finally, the sections were sealed and mounted with neutral resin.

#### Immunofluorescence Staining

Briefly, fresh tissues were immersed in 4% paraformaldehyde (Sigma-Aldrich) for fixation at room temperature for 30 min. The tissues were then dehydrated in an ethanol gradient, embedded in paraffin, sectioned (thickness: 6 μm), and immersed in xylene for dewaxing. Tissue sections were blocked with immunohistochemical blocking solution (Beyotime Biotechnology Co., Ltd., Zhejiang, China) at 37 °C for 30 min. The blocking solution was then discarded, and the sections were washed three times at room temperature for 5 min, each with immunohistochemical washing solution (Beyotime Biotechnology). Then, primary antibodies (rabbit anti-GPX4 antibody [EPNCIR144] (ab125066), rabbit anti-ferritin heavy chain antibody [EPR18878] (ab183781), and rabbit anti-Ki67 antibody (ab15580), Abcam, MA, USA) were added and incubated at 37 °C for 45 min. After incubation, the antibody solution was discarded, and the sections were washed three times at room temperature for 5 min each with immunohistochemical washing solution (Beyotime Biotechnology). Then, the secondary antibody (goat anti-rabbit IgG H&L (Alexa Fluor 488), Abcam, MA, USA) was added, and the tissues were incubated at 37 °C for 45 min. After incubation, the antibody solution was discarded, and the sections were washed three times at room temperature for 5 min, each with immunohistochemical washing solution (Beyotime Biotechnology). Finally, immunofluorescence blocking solution (Sigma-Aldrich) was added, and the sections were mounted.

#### RNA Extraction, RNA Extraction with Reverse Transcription Reaction, and qPCR

According to the instructions of the RNAprep pure Tissue Kit (TIANGEN Biotech (Beijing) China) Co., Ltd., Beijing, China), to about 20 mg of human tissue specimen was added 800 μL of lysis solution, and the mixture was ground, homogenized, and centrifuged. To the supernatant, 200 μL of chloroform was added, and the mixture was mixed by inverting and centrifuged at 4 °C at 13,400 × *g* for 15 min. To the supernatant, two volumes of absolute ethanol were added, mixed by inversion, and centrifuged at 4 °C at 13,400 × *g* for 30 min. Ethanol precipitation and centrifugation of the supernatant were repeated. The RNA pellet was suspended with 500 μL of 75% ethanol and centrifuged at 13,400 × *g* at 4 °C for 5 min. The supernatant was discarded, the excess liquid was removed, and the pellet was dissolved in 300 μL of DECP water. One microliter of the RNA solution was used to measure the ratio of *A*_260_–*A*_280_ (generally 1.8–2.0) to determine the purity and approximate concentration of the RNA. RNA samples were treated with DNase I (Sigma-Aldrich), requantified, and reverse-transcribed into cDNA using the ReverTra Ace-α First Strand cDNA Synthesis Kit (Toyobo). Quantitative real-time PCR (qPCR) was conducted using a RealPlex4 real-time PCR detection system (Eppendorf, Germany) with SYBR Green Realtime PCR Master Mix (Toyobo). On a quantitative real-time PCR instrument, the following reactions were performed: 95 °C for 15 min; 94 °C for 20 s; 60 °C for 34 s, and the fluorescence value was read. The above reactions were performed for 40 cycles. The qPCR primers were described previously.^69,70^

#### In Vitro Oxidative Metabolic Stability

##### Intrinsic Clearance in Microsomes^71^

Mouse (Sigma Aldrich, CD-1 male, pooled) and human microsomes (Sigma Aldrich, human, pooled) at 0.5 mg/mL were preincubated with the test compound **15** dissolved in DMSO at 1 μM in phosphate buffer 50 mM, pH 7.4, and 3 mM MgCl_2_ for 10 min at 37 °C. The reaction was then started by adding the cofactor mixture solution (NADP, glucose-6-phosphate, glucose-6-phosphate dehydrogenase in 2% NaHCO_3_). Samples were taken at 0, 10, 30, 45, and 60 min and added to acetonitrile to stop the reaction. Samples were then centrifuged, and the supernatant was analyzed by LC–MS/MS to quantify the amount of the compound. A control sample without the cofactor was always added to check the chemical stability of the test compound. Two reference compounds of known metabolic stability, 7-EC and propranolol, were present in parallel testing. A fixed concentration of verapamil was added in every sample as an internal standard for LC–MS/MS analyses. The percentage of the area of the test compound remaining at the various incubation times was calculated with respect to the area of the compound at time 0 min.

The intrinsic clearance (Cli) was calculated by the following equation:
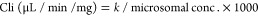
where *k* is the rate constant (min^–1^); microsomal protein conc. = 0.5 mg protein/mL. The rate constant, *k* (min^–1^), derived for the exponential decay equation (peak area/IS vs time), was used to calculate the rate of Cli. Classification of in vitro stability is presented in Table 4.

#### LC–MS/MS Analytical Method

Samples were analyzed under the following conditions: UPLC Waters coupled with an API 3200 triple-quadrupole (ABSciex); eluents, (phase A) 95% water, 5% acetonitrile + 0.1% HCOOH, (phase B) 5% water, 95% acetonitrile + 0.1% HCOOH; flow rate, 0.3 mL/min; column, Gemini-Nx 5 μm C18 110A (50 × 2.00 mm) at 35 °C; injection volume, 10 μL. Source conditions ESI positive: T 400 °C, Gas 1 30, Gas 2 35, CUR 30, IS 5500, CAD 5.

## Supporting Information Available

Additional biological information on the treatment of human primary T cells with **15** (Figures 1 SI and 2 SI); chromatographic analyses (Figures 3S–17S); and relative area (%) recorded at 254 nm (Table S1) of compound **2–16** (PDF)
Molecular formula strings with the associated biological data (CSV)

## Notes

^∇^ M.P. and J.W. are the first co-authors.

## Abbreviations

ARDHEP: diheterocyclic pyrrole
EC: ethoxycoumarin
GBM: glioblastoma multiforme
GPX4: glutathione peroxidase 4
GSH: glutathione
GSSG: oxidized glutathione
HE: hematoxylin and eosin
LPO: lactoperoxidase
MCF-7: human breast carcinoma
MDA: malondialdehyde
OC: ovarian cancer
OVCAR-3: ovarian adenocarcinoma cells
qPCR: quantitative polymerase chain reactions
SDS: sodium dodecyl sulfate
T-GSH: total glutathione
U-87 MG: human glioblastoma cells
