# Supplementary material for: Induction of Ferroptosis in Glioblastoma and Ovarian Cancers by a New Pyrrole Tubulin Assembly Inhibitor
Source: J Med Chem. 2022 Nov 17;65(23):15805–18. doi: 10.1021/acs.jmedchem.2c01457 (PMC9743090; doi:10.1021/acs.jmedchem.2c01457)
Supplement: Supplementary file 1 — jm2c01457_si_001.pdf [file jm2c01457_si_001.pdf]

Supporting Information

Induction of Ferroptosis in Glioblastoma and Ovarian Cancers by a New Pyrrole Tubulin Assembly Inhibitor

Michela Puxeddu,[†,#] Jianchao Wu,[§,#] Ruoli Bai,[‡] Michele D'Ambrosio,[†] Marianna Nalli,[†] Antonio Coluccia,[†] Simone Manetto,[†] Alessia Ciogli,[†] Domiziana Masci,[£] Andrea Urbani,[£] Cinzia Fionda,[¢] Sonia Coni,[¢] Rosa Bordone,[¢] Gianluca Canettieri,[¢] Chiara Bigogno,[f] Giulio Dondio,[f] Ernest Hamel,[‡] Te Liu,[§,*] Romano Silvestri,[†,*] Giuseppe La Regina,[†,*]

[†]Laboratory Affiliated with the Institute Pasteur Italy - Cenci Bolognetti Foundation, Department of Drug Chemistry and Technologies, Sapienza University of Rome, Piazzale Aldo Moro 5, 00185 Rome, Italy

[‡]Molecular Pharmacology Branch, Developmental Therapeutics Program, Division of Cancer Treatment and Diagnosis, Frederick National Laboratory for Cancer Research, National Cancer Institute, National Institutes of Health, Frederick, Maryland 21702, United States

[£]Department of Basic Biotechnological Sciences, Intensivological and Perioperative Clinics, Catholic University of the Sacred Heart, Largo Francesco Vito 1, 00168 Rome, Italy

[¢]Laboratory Affiliated with the Institute Pasteur Italy - Cenci Bolognetti Foundation, Department of Molecular Medicine, Sapienza University of Rome, Viale Regina Elena 291, 00161 Rome, Italy

[f]Aphad SrL, Via della Resistenza 65, 20090 Buccinasco, Italy

[§]Shanghai Geriatric Institute of Chinese Medicine, Shanghai University of Traditional Chinese Medicine, 365 South Xiangyang Road, 200031 Shanghai, China

#First co-author: M.P.; J.W.:
*Corresponding Authors
T.L.: E-Mail: liute1979@shutcm.edu.cn; R.S.: E-mail: romano.silvestri@uniroma1.it; G.L.R.: E-mail: giuseppe.laregina@uniroma1.it.

Contents of SI

Figure S1, page S2, Treatment of human primary T cells with **15**.
Figure S2, page S3, Percentage of apoptotic cells and dead cells upon treatment of human primary T cells with **15**.
Figure S3, page S3, Chromatographic analyses of compound **2**.
Figure S4, page S4, Chromatographic analyses of compound **3**.
Figure S5. page S4, Chromatographic analyses of compound **4**.
Figure S6, page S5, Chromatographic analyses of compound **5**.
Figure S7, page S5**.** Chromatographic analyses of compound **6**.
Figure S8, page S6**,** Chromatographic analyses of compound **7**.
Figure S9, page S6. Chromatographic analyses of compound **8**.
Figure S10, page S7. Chromatographic analyses of compound **9**.
Figure S11, page S7. Chromatographic analyses of compound **10**.
Figure S12. page S8. Chromatographic analyses of compound **11**.
Figure S13, page S8**,** Chromatographic analyses of compound **12**.
Figure S14, page S9**,** Chromatographic analyses of compound **13**.
Figure S15, page S9**,** Chromatographic analyses of compound **14**.
Figure S16, page S10**,** Chromatographic analyses of compound **15**.
Figure S17, page S10**,** Chromatographic analyses of compound **16**.
Table S1 Relative area (%) recorded at 254 nm.

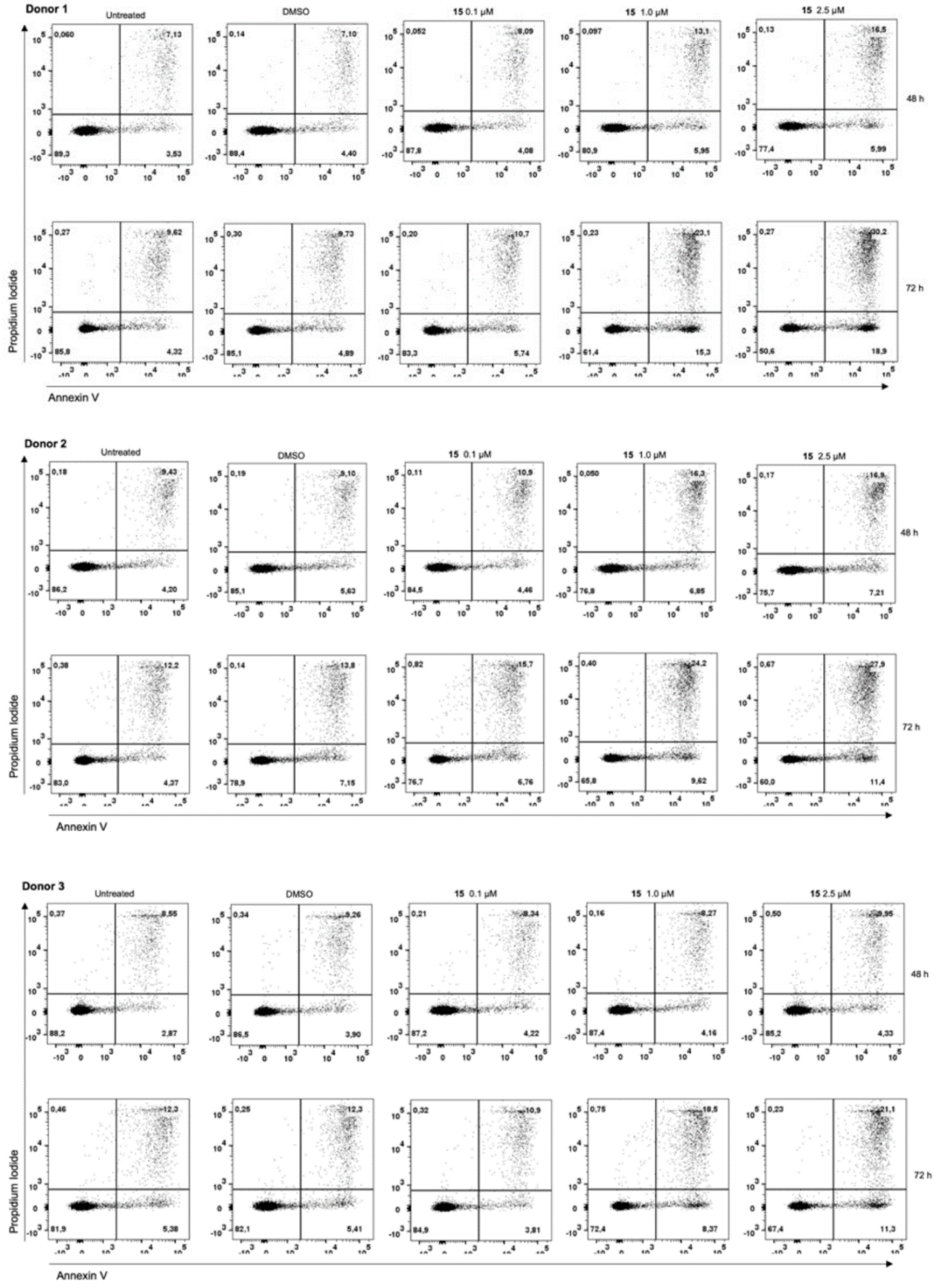

Figure S1. Human primary T cells were treated with the indicated concentrations of compound **15** or DMSO for 48 or 72 h and analyzed for annexin V by flow cytometry. The frequency of annexin-V single positive cells (lower right, early apoptotic cells) and annexin V/propidium iodide double positive cells (upper right, late phase apoptotic cells) is shown. Data from two different healthy donors (Donors 1, 2 and 3) are shown.

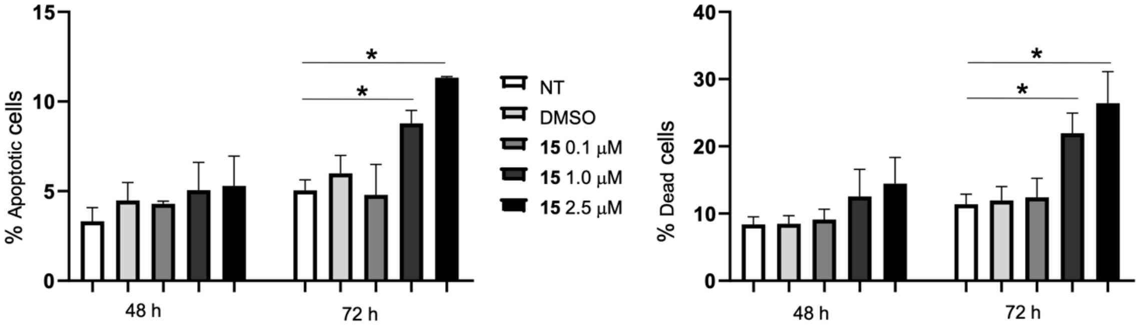

Figure S2. Percentage of apoptotic cells (left panel) and dead cells (right panel) upon treatment of human primary T cells with the indicated concentrations of **15** or DMSO for 48 or 72 h.

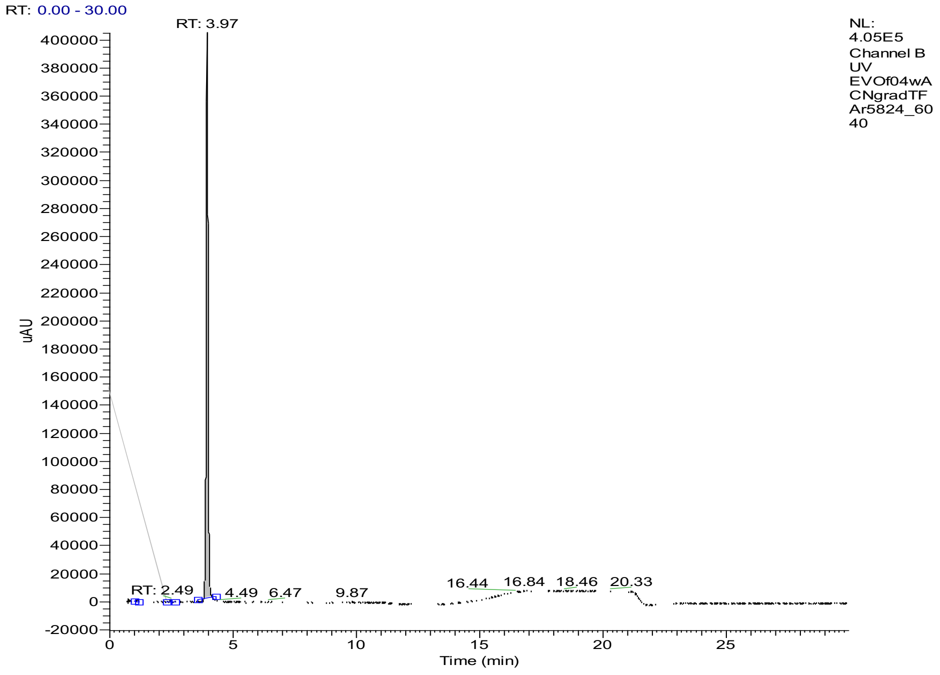

Figure S3 Chromatographic analysis of compound **2**. Mobile Phases: A= Water + TFA 0.1 % v/v. B = ACN/Water 95:5 + TFA 0.1 % v/v. Gradient elution: 40 % B (10 min), 90% B (15 min), 90% B (20 min), 40 % B (21 min), 40 % B (30 min). Sample solved in A/B 1:1.

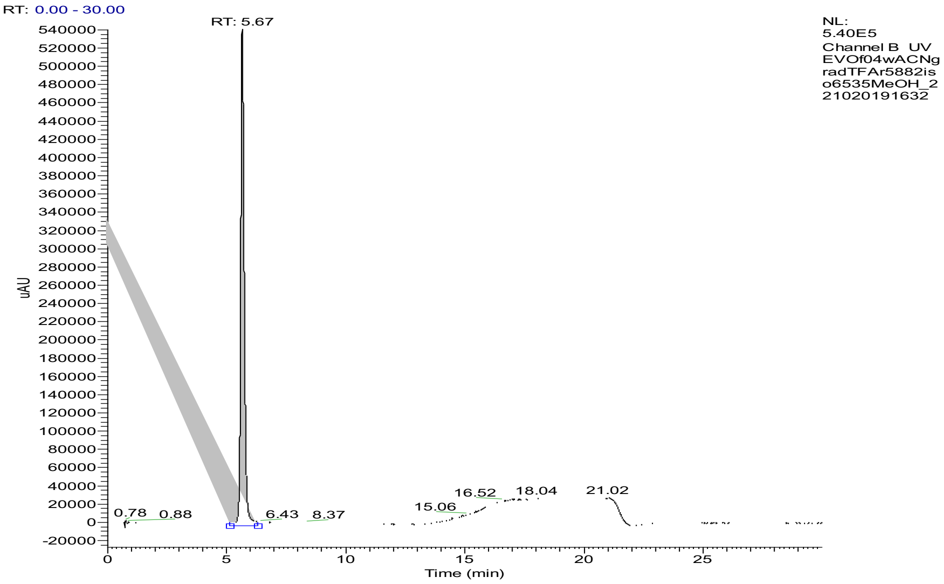

Figure S4 Chromatographic analysis of compound **3**. Mobile Phases: A= Water + TFA 0.1 % v/v. B = MeOH + TFA 0.1 % v/v. Gradient elution: 35 % B (10 min), 90% B (15 min), 90% B (20 min), 35 % B (21 min), 35 % B (30 min). Sample solved in A/B 1:1.

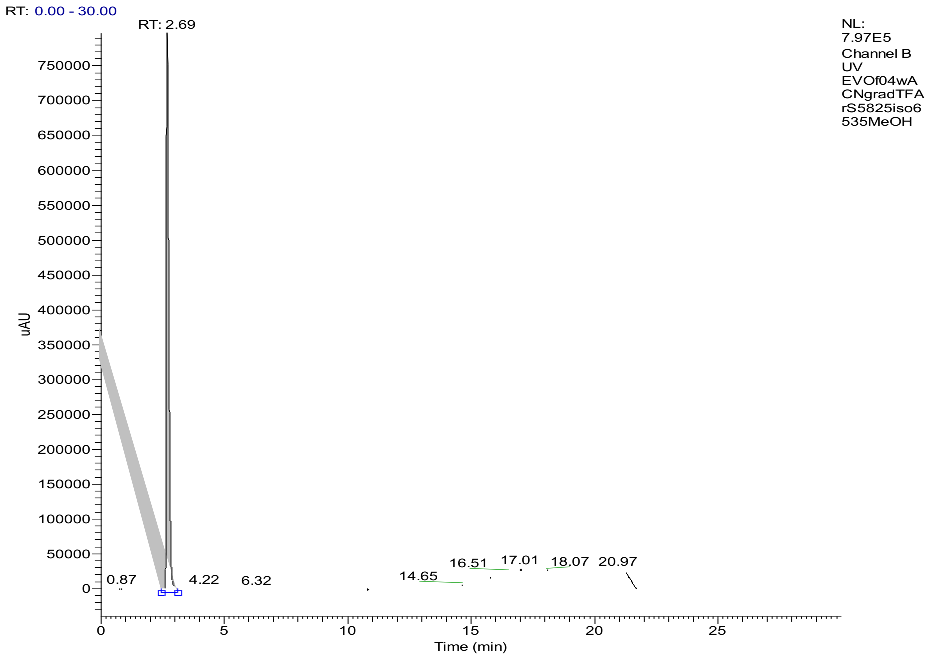

Figure S5 Chromatographic analysis of compound **4**. Mobile Phases: A= Water + TFA 0.1 % v/v. B = MeOH + TFA 0.1 % v/v. Gradient elution: 35 % B (10 min), 90% B (15 min), 90% B (20 min), 35 % B (21 min), 35 % B (30 min). Sample solved in A/B 1:1.

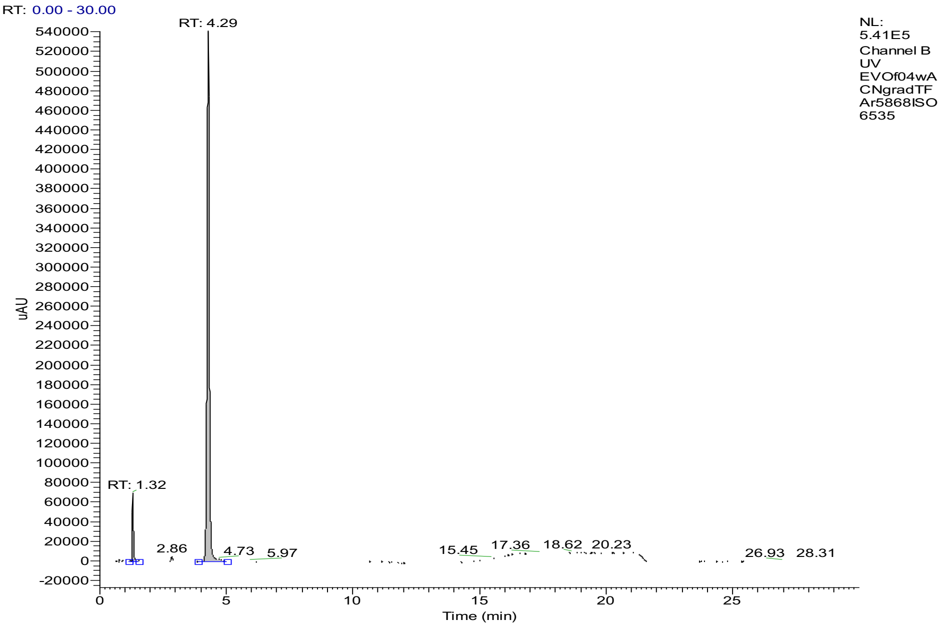

Figure S6 Chromatographic analysis of compound **5**. Mobile Phases: A= Water/ACN 95:5 + TFA 0.1 % v/v. B = ACN/Water 95:5 + TFA 0.1 % v/v. Gradient elution: 35 % B (10 min), 90% B (15 min), 90% B (20 min), 35 % B (21 min), 35 % B (30 min). Sample solved in A/B 1:1.

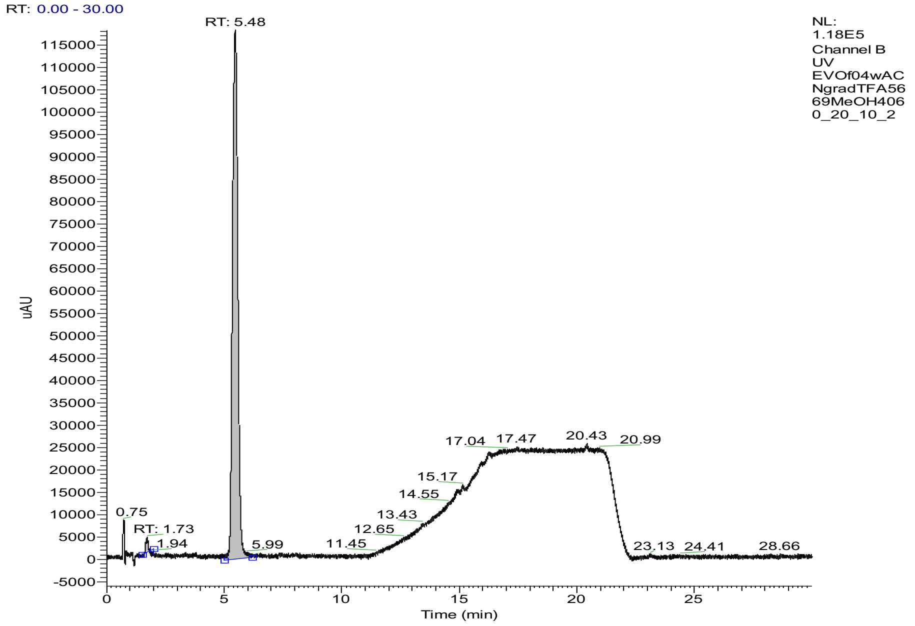

Figure S7. Chromatographic analysis of the compound **6**. Mobile Phases: A= Water + TFA 0.1 % v/v. B = MeOH + TFA 0.1 % v/v. Gradient elution: 60 % B (10 min), 90% B (15 min), 90% B (20 min), 60 % B (21 min), 60 % B (30 min). Sample solved in A/B 1:1.

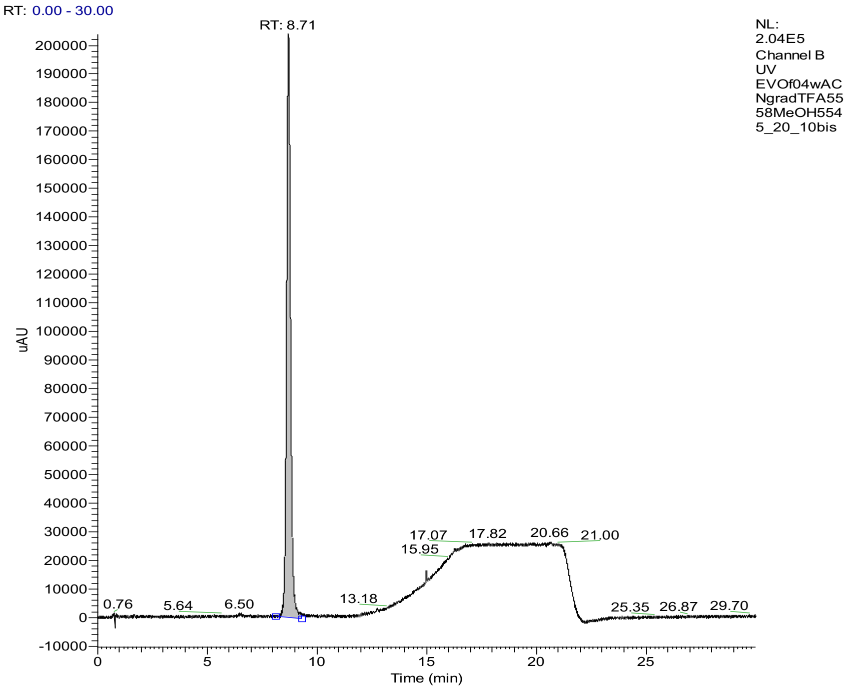

Figure S8 Chromatographic analysis of compound **7**. Mobile Phases Mobile Phases: A= Water + TFA 0.1 % v/v. B = MeOH + TFA 0.1 % v/v. Gradient elution: 45 % B (10 min), 90% B (15 min), 90% B (20 min), 45 % B (21 min), 45 % B (30 min). Sample solved in A/B 1:1.

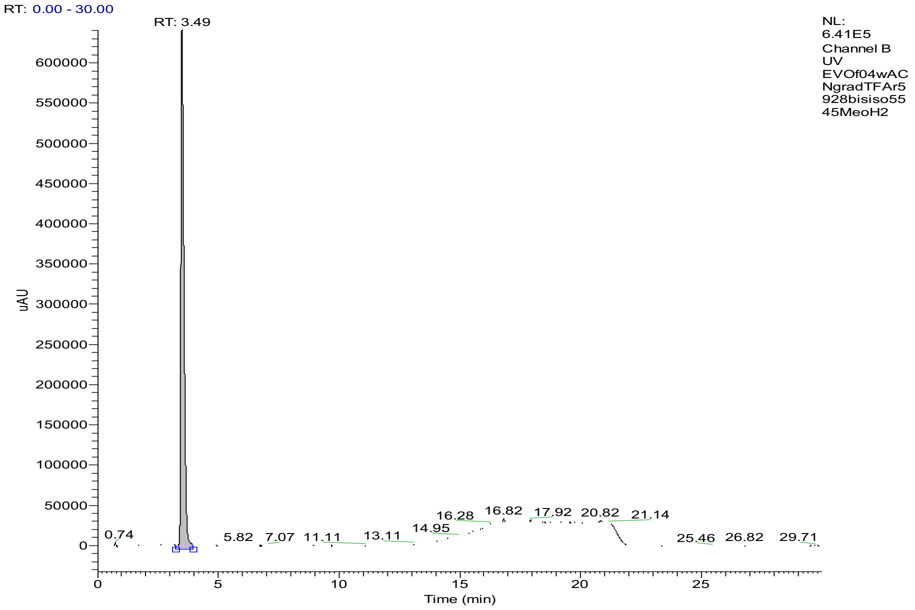

Figure S9. Chromatographic analysis of compound **8**. Mobile Phases: A= Water + TFA 0.1 % v/v. B = MeOH + TFA 0.1 % v/v. Gradient elution: 45 % B (10 min), 90% B (15 min), 90% B (20 min), 45 % B (21 min), 45 % B (30 min). Sample solved in A/B 1:1.

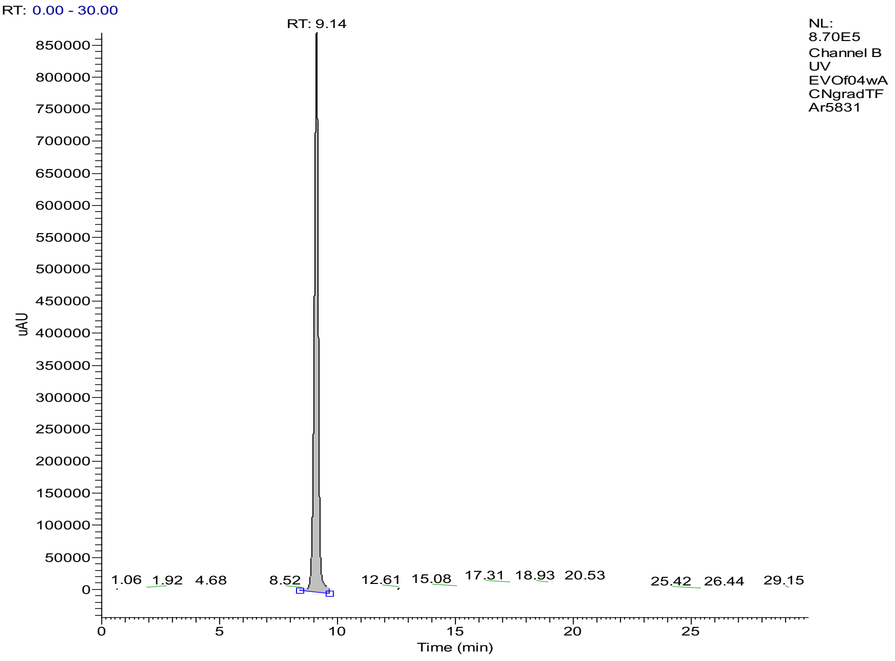

Figure S10. Chromatographic analysis of tcompound **9**. Mobile Phases: A= Water/ACN 95:5 + TFA 0.1 % v/v. B = ACN/Water 95:5 + TFA 0.1 % v/v. Gradient elution: 40 % B (10 min), 90% B (15 min), 90% B (20 min), 40 % B (21 min), 40 % B (30 min). Sample solved in A/B 1:1.

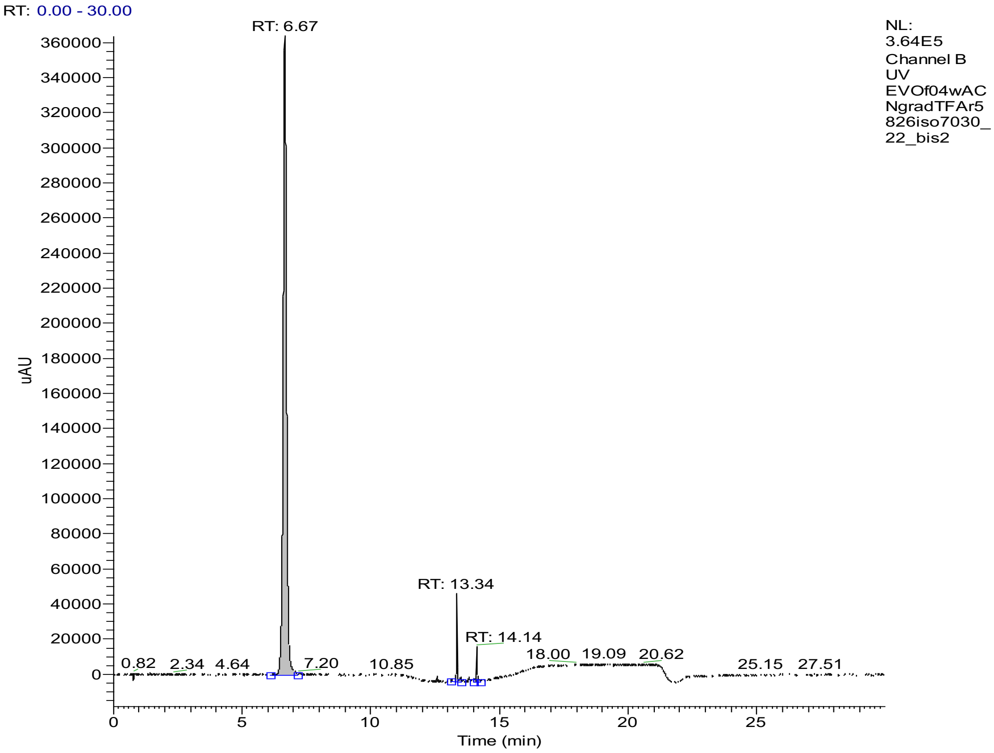

Figure S11. Chromatographic analysis of compound **10**. Mobile Phases: Mobile Phases: A= Water + TFA 0.1 % v/v. B = ACN/Water 95:5 + TFA 0.1 % v/v. Gradient elution: 30 % B (10 min), 90% B (15 min), 90% B (20 min), 30 % B (21 min), 30 % B (30 min). Sample solved in A/B 1:1.

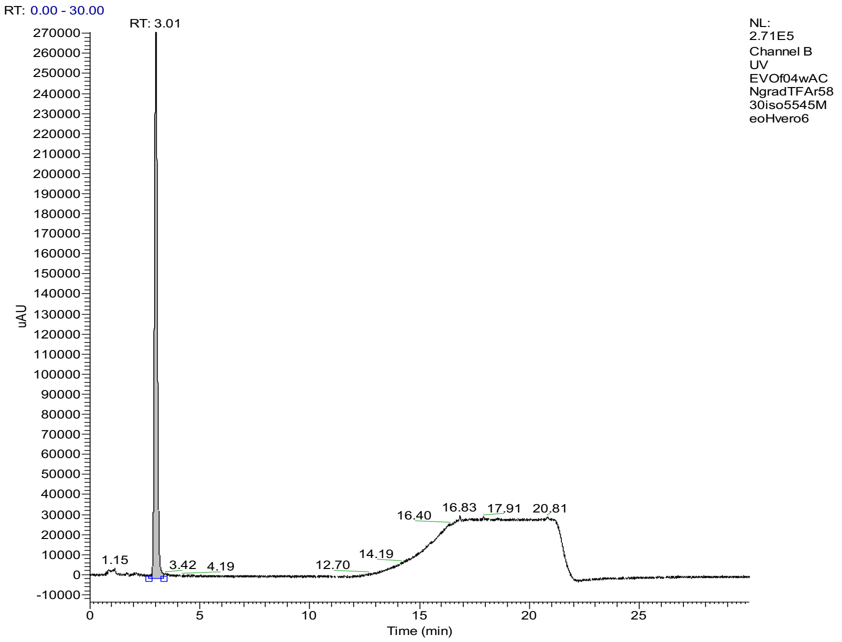

Figure S12. Chromatographic analysis of compound **11.** Mobile Phases: A= Water + TFA 0.1 % v/v. B = MeOH + TFA 0.1 % v/v. Gradient elution: 45 % B (10 min), 90% B (15 min), 90% B (20 min), 45 % B (21 min), 45 % B (30 min). Sample solved in A/B 1:1.

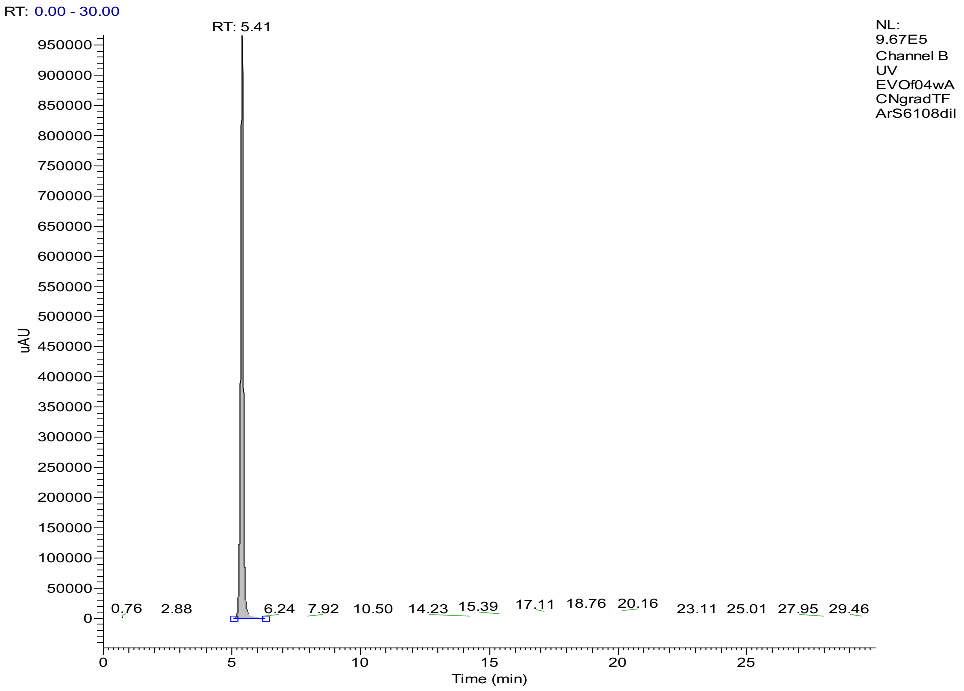

Figure S13 Chromatographic analysis of compound **12**. Mobile Phases: A= Water/ACN 95:5 + TFA 0.1 % v/v. B = ACN/Water 95:5 + TFA 0.1 % v/v. Gradient elution: 40 % B (10 min), 90% B (15 min), 90% B (20 min), 40 % B (21 min), 40 % B (30 min). Sample solved in A/B 1:1.

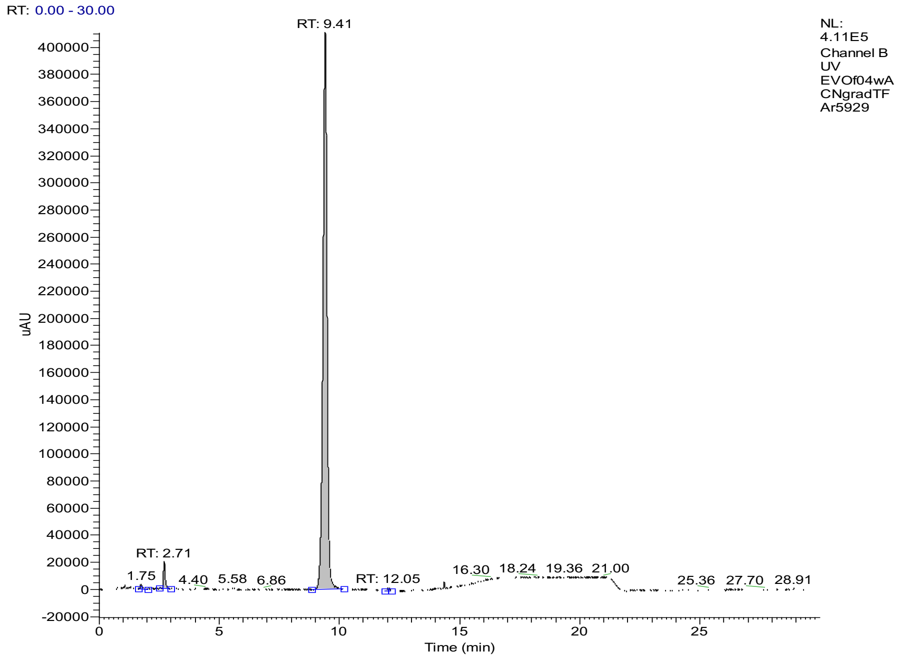

Figure S14 Chromatographic analysis of compound **13**. Mobile Phases: A= Water/ACN 95:5 + TFA 0.1 % v/v. B = ACN/Water 95:5 + TFA 0.1 % v/v. Gradient elution: 40 % B (10 min), 90% B (15 min), 90% B (20 min), 40 % B (21 min), 40 % B (30 min). Sample solved in A/B 1:1.

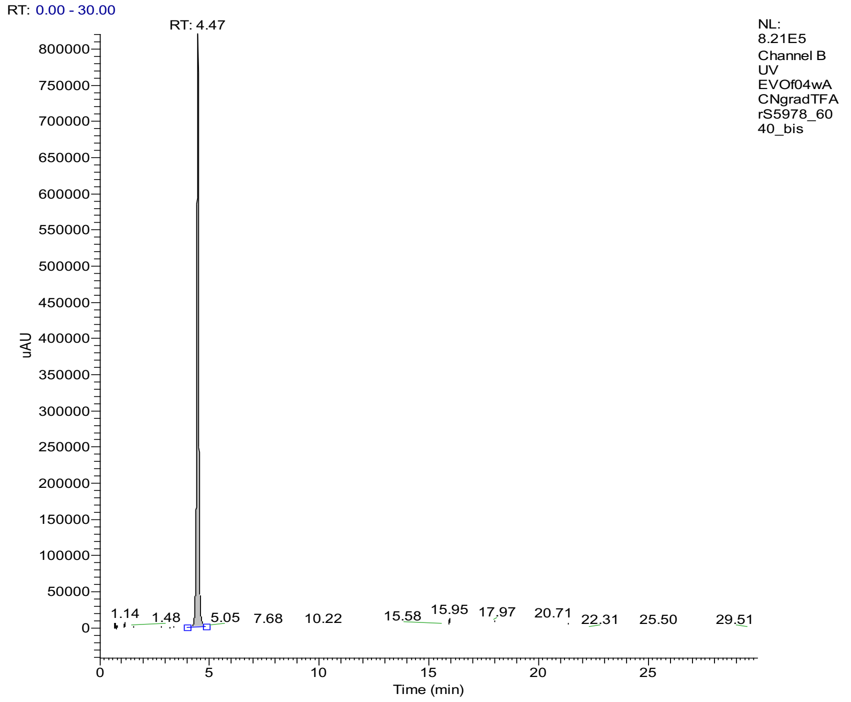

Figure S15 Chromatographic analysis of compound **14**. Mobile Phases: A= Water + TFA 0.1 % v/v. B = ACN/Water 95:5 + TFA 0.1 % v/v. Gradient elution: 40 % B (10 min), 90% B (15 min), 90% B (20 min), 40 % B (21 min), 40 % B (30 min). Sample solved in A/B 1:1.

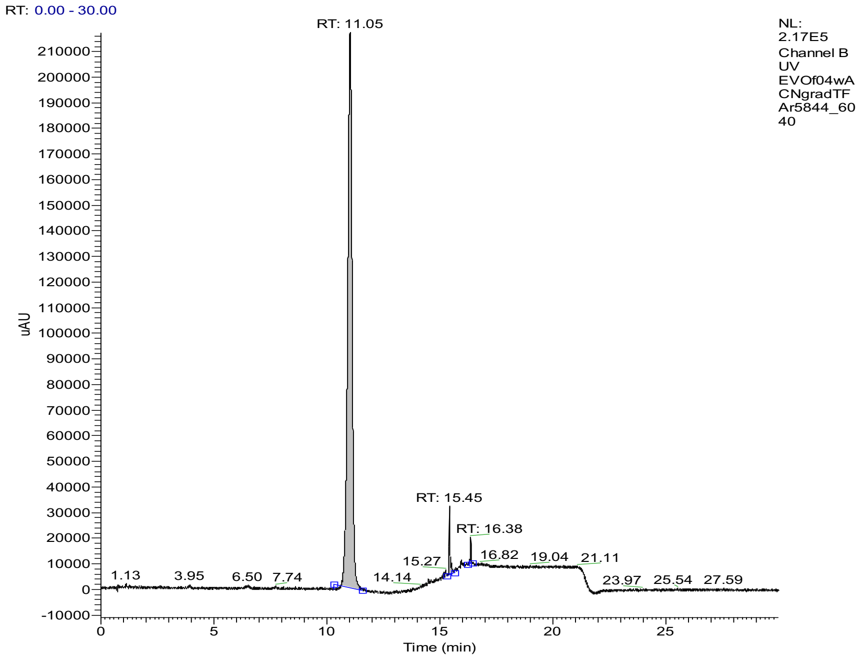

Figure S16 Chromatographic analysis of compound **15**. Mobile Phases: A= Water + TFA 0.1 % v/v. B = ACN/Water 95:5 + TFA 0.1 % v/v. Gradient elution: 40 % B (10 min), 90% B (15 min), 90% B (20 min), 40 % B (21 min), 40 % B (30 min). Sample solved in A/B 1:1.

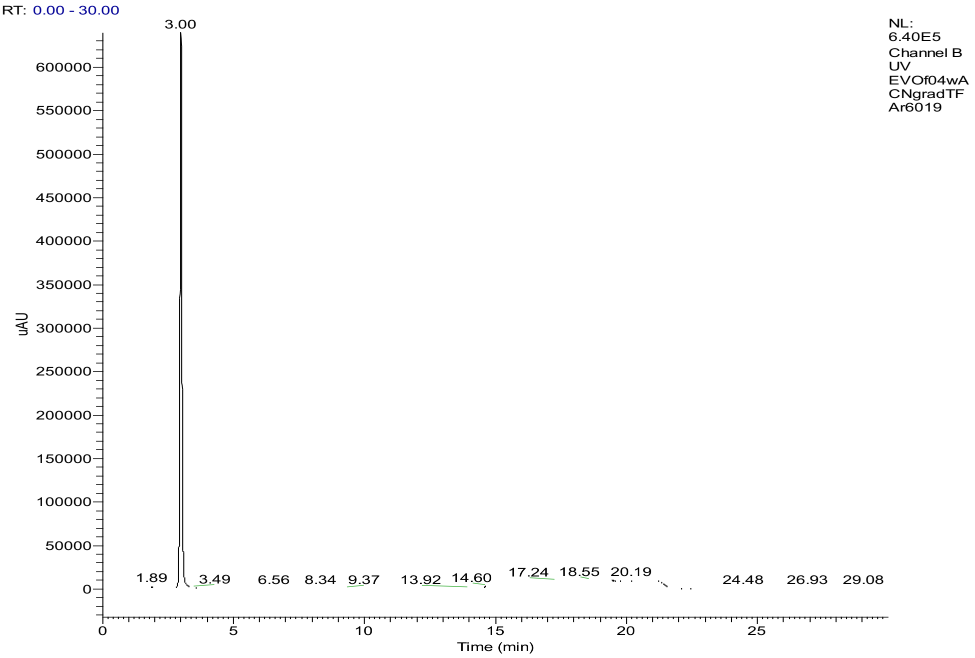

Figure S17 Chromatographic analysis of compound **16**. Mobile Phases: A= Water/ACN 95:5 + TFA 0.1 % v/v. B = ACN/Water 95:5 + TFA 0.1 % v/v. Gradient elution: 40 % B (10 min), 90% B (15 min), 90% B (20 min), 40 % B (21 min), 40 % B (30 min). Sample solved in A/B 1:1.

Table S1 Relative area (%) recorded at 254 nm.

Compd	254 nm Rel. Area (%)
**2**	99.23
**3**	>99.99
**4**	>99.99
**5**	95.05
**6**	98.86
**7**	>99.99
**8**	>99.99
**9**	>99.99
**10**	95.75
**11**	>99.99
**12**	>99.99
**13**	95.96
**14**	>99.99
**15**	96.54
**16**	99.36
